# Differential gene expression analysis based on linear mixed model corrects false positive inflation for studying quantitative traits

**DOI:** 10.1038/s41598-023-43686-7

**Published:** 2023-10-03

**Authors:** Shizhen Tang, Aron S. Buchman, Yanling Wang, Denis Avey, Jishu Xu, Shinya Tasaki, David A. Bennett, Qi Zheng, Jingjing Yang

**Affiliations:** 1 Department of Human Genetics, Center for Computational and Quantitative Genetics, Emory University School of Medicine, Atlanta, GA 30322 USA; 2 Department of Biostatistics and Bioinformatics, Emory University School of Public Health, Atlanta, GA 30322 USA; 3 Rush Alzheimer’s Disease Center, Rush University Medical Center, Chicago, IL 60612 USA; 4 Department of Bioinformatics and Biostatistics, University of Louisville, 485 E. Gray St, Louisville, KY 40202 USA

**Keywords:** Statistical methods, Gene expression, Quantitative trait, RNAi

## Abstract

Differential gene expression (DGE) analysis has been widely employed to identify genes expressed differentially with respect to a trait of interest using RNA sequencing (RNA-Seq) data. Recent RNA-Seq data with large samples pose challenges to existing DGE methods, which were mainly developed for dichotomous traits and small sample sizes. Especially, existing DGE methods are likely to result in inflated false positive rates. To address this gap, we employed a linear mixed model (LMM) that has been widely used in genetic association studies for DGE analysis of quantitative traits. We first applied the LMM method to the discovery RNA-Seq data of dorsolateral prefrontal cortex (DLPFC) tissue (*n* = 632) with four continuous measures of Alzheimer’s Disease (AD) cognitive and neuropathologic traits. The quantile–quantile plots of *p*-values showed that false positive rates were well calibrated by LMM, whereas other methods not accounting for sample-specific mixed effects led to serious inflation. LMM identified 37 potentially significant genes with differential expression in DLPFC for at least one of the AD traits, 17 of which were replicated in the additional RNA-Seq data of DLPFC, supplemental motor area, spinal cord, and muscle tissues. This application study showed not only well calibrated DGE results by LMM, but also possibly shared gene regulatory mechanisms of AD traits across different relevant tissues.

Next-generation sequencing technology has been widely used in genetics and genomics studies to elucidate the biology underlying complex human diseases and traits^1^. RNA sequencing (RNA-Seq) technology has been widely used to profile transcriptome-wide gene expression levels and has revolutionized transcriptome analyses^2,3^. Differential gene expression (DGE) analysis is one approach for studying RNA-Seq data to identify genes expressed differentially with respect to a trait of interest^3–5^. Due to the cost of RNA-Seq studies and the difficulty in obtaining large numbers of relevant tissue samples from individuals, most existing DGE methods have been developed to handle small sample sizes and dichotomous traits, e.g., DESeq2^6^, edgeR^7,8^, Limma^9^, Voom^10^, and MACAU^11^. However, with the recently reduced cost of RNA-Seq technology, RNA-Seq data from hundreds of samples have been generated for studying both dichotomous and continuous quantitative traits.

Existing DGE methods generally need to dichotomize continuous phenotypes, thus failing to account for the continuous distribution of quantitative traits^6–9,11^. As a result, information could be lost, and power could be reduced by not characterizing the continuous characteristics of phenotypes. This loss of information by dichotomizing a continuous biologic process (i.e., a continuous trait) may homogenize individuals together who often, in fact, lie on a continuum of disease, especially for chronic conditions of aging. For example, the cognitive manifestation of AD related dementia unfolds over years to decades^12^. Further, in older persons, AD dementia is often due to a combination of mixed pathologies and resilience^13,14^, which is better characterized by continuous cognitive traits and neuropathologic traits. During the prolonged course of this chronic disease, individuals who initially show no cognitive impairment (NCI) may manifest mild cognitive impairment (MCI) for years before they finally develop Alzheimer’s dementia as a late final manifestation (Supplemental Table 1)^15^. Moreover, these categories themselves are not distinct but represent stages along a progressive continuum.

**Table 1:** Clinical and postmortem characteristics of the discovery analytic cohort.

Traits	Variable (range)	Mean (SD) or N (%)
Demographics	Age at death (years) (67.4, 108.3)	88.6 (6.65)
	Male	215 (36.3%)
	Postmortem Interval (PMI, hours) (1, 40.8)	7.3 (4.88)
	MAP participants	283 (47.8)
Clinical AD Trait	Rate of cognitive decline ( − 0.42, 0.14)	− 0.02 (0.1)
AD Neuropathologic Changes (AD-NC)	β-Amyloid (0.00, 19.93)	3.96 (4.13)
	Tangle density (0.00, 78.52)	6.2 (7.6)
	Global AD pathology burden (0.00, 3.21)	0.68 (0.6)

The decreasing cost of RNA-Seq technology and the recognition of the importance of RNA-seq data have led to the recent availability of much larger RNA-Seq sample sizes from individuals with both dichotomous and quantitative phenotypes^16–18^. For example, the Genotype-Tissues Expression (GTEx) project V8 profiled hundreds of samples per tissue for 53 human tissues (up to *n* = 803 for muscle tissue)^19^; the CommonMind Consortium sequenced RNA from dorsolateral prefrontal cortex (DLPFC) of people with schizophrenia (*n* = 258) and control subjects (*n* = 279) for studying schizophrenia and other psychological diseases^20^; the prospective cohort studies of Religious Orders Study (ROS) and the Rush Memory and Aging Project (MAP) sequenced RNA from DLPFC of ~ 1200 participants for study AD traits including the continuous cognitive decline and continuous markers of AD neuropathologic changes (AD-NC), i.e., neurofibrillary tangles (NFTs) and beta amyloid (Aβ))^17^. Enabling DGE of continuous quantitative traits (such as cognitive decline and AD-NC traits) with hundreds of sample sizes is crucial to advance our understanding and the development of targeted treatments for complex diseases.

Recently, methods based on the standard linear regression^21^ and robust regression^19,21^ were proposed for DGE analysis of quantitative traits, which takes the quantitative trait as the response variable and the log2 transformed RNA-Seq read counts per gene as the test covariate^21^ (see Methods). However, the methods based on standard linear regression and robust regression models often lead to inflated false positive rates by failing to account for unknown confounders^11,22,23^. To improve on existing DGE methods, we apply the linear mixed model (LMM) based method as implemented by the Genome-wide Efficient Mixed Model Association (GEMMA) tool^22^ to conduct DGE of quantitative traits. The LMM based method can account for shared confounding factors among test samples through the sample-specific mixed effect term, which has been widely used in large-scale genetic association testing to achieve calibrated false positive rates^22–24^. The Linear Mixed Model (LMM) implemented by GEMMA employs the full-rank sample-sample correlation matrix (based on all gene expressions) to model the sample-specific random effects (see Methods), which models unknown confounding factors and thus corrects the inflated false positives that occur in linear regression models without mixed effect terms^22^. To demonstrate the feasibility of this approach, we developed an analytic LMM pipeline to conduct DGE, and applied the pipeline to study four cognitive and pathologic AD traits—the rate of cognitive decline and three AD-NC traits (*β*-amyloid, tangle density, global AD pathology burden).

We first used the LMM pipeline to conduct DGE analysis using discovery RNA-Seq data of DLPFC brain tissue (*n* = 632) with respect to cognitive decline and each of the AD-NC traits. Several previous studies have shown that Alzheimer Disease affects not only cognition but also non-cognitive traits such as motor functions that may be affected by the accumulation of AD-NC in tissues outside the brain^25^. Thus, to validate our findings with the discovery RNA-Seq data of DLPFC, we then conducted DGE analysis on the same continuous cognitive decline and AD-NC traits using additional RNA-Seq datasets from DLPFC brain tissue (*n* = 588) and three tissues relevant to motor functions a) supplementary motor area (SMA) within the brain (*n* = 234), (b) lumbar spinal cord (*n* = 232) neural tissues within the central nervous system (CNS) but outside the brain, and (c) muscle (*n* = 268), the final effector of all volitional movement, composted of non-neural tissue and located in the periphery outside the CNS. We showed that false positive rates were well calibrated by the LMM based method, compared to serious inflation of the DGE results by using the standard linear regression^21^, robust regression^21^, and Voom^10^. As a result, our DGE analyses by LMM identified 37 genes differentially expressed for either cognitive decline or at least one of the AD-NC traits, with 17 of those genes replicated in the additional RNA-Seq data from DLPFC, SMA, spinal cord, and muscle tissues.

## Results

### DGE in the discovery data of DLPFC tissue

**Figure 1:**
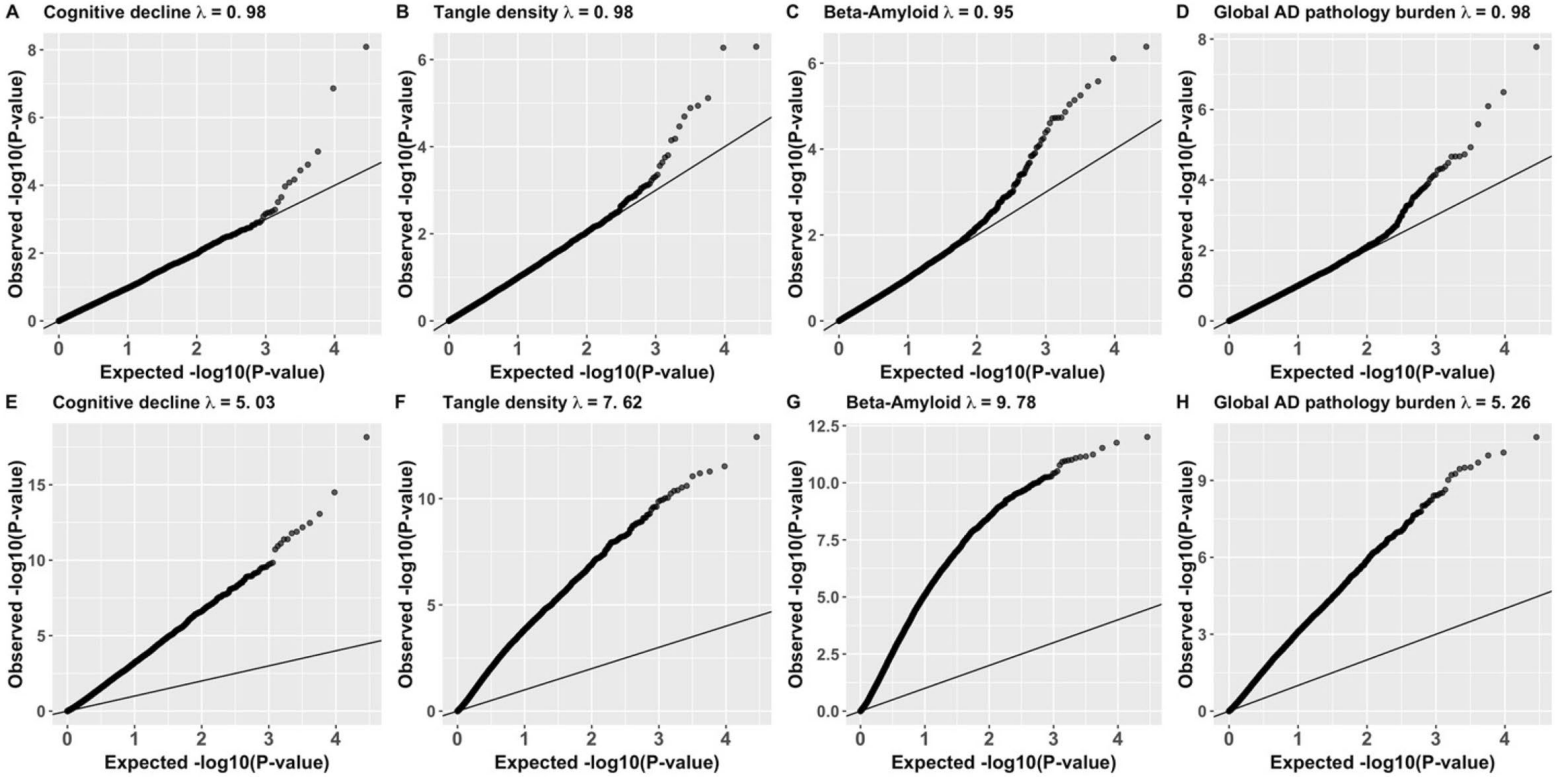
QQ-plots and genomic control factors of DGE results by LMM (**A**–**D**) and standard linear regression model (**E**–**H**) with the discovery RNA-Seq data of DLPFC tissue of cognitive decline and three AD-NC traits.

We compared the results obtained from the DGE analyses of continuous cognitive decline and three AD-NC traits, by using four methods: LMM, standard linear regression model, robust regression, and Voom with quantitative traits dichotomized by their medians (see Methods). Our DGE analyses adjusted for various covariates, including sex, age, postmortem interval (PMI, time span between donor’s death and tissue harvest) and study group (ROS or MAP) in both models (Table 1). We constructed Quantile–Quantile plots (QQ-plots) to visualize the DGE p-values of all test genes per trait, and calculated the genomic control factor $\lambda$.^26^ As depicted in Fig. 1, LMM-based test method well calibrated false positive rates (with $\lambda \sim 1$) for all traits (First Row in Fig. 1) with the discovery RNA-seq data of DLPFC tissue, while the standard linear regression method resulted in seriously inflated false positive rates associated (with $\lambda >5$) for all four traits (Second Row in Fig. 1). High inflated false positive rates were also observed in the results of all four traits with the discovery data by using the robust regression method (First Row in Supplemental Fig. 1) with $\lambda >3$, and by using the Voom method (First Row in Supplemental Fig. 2) with $\lambda >2$.

**Figure 2:**
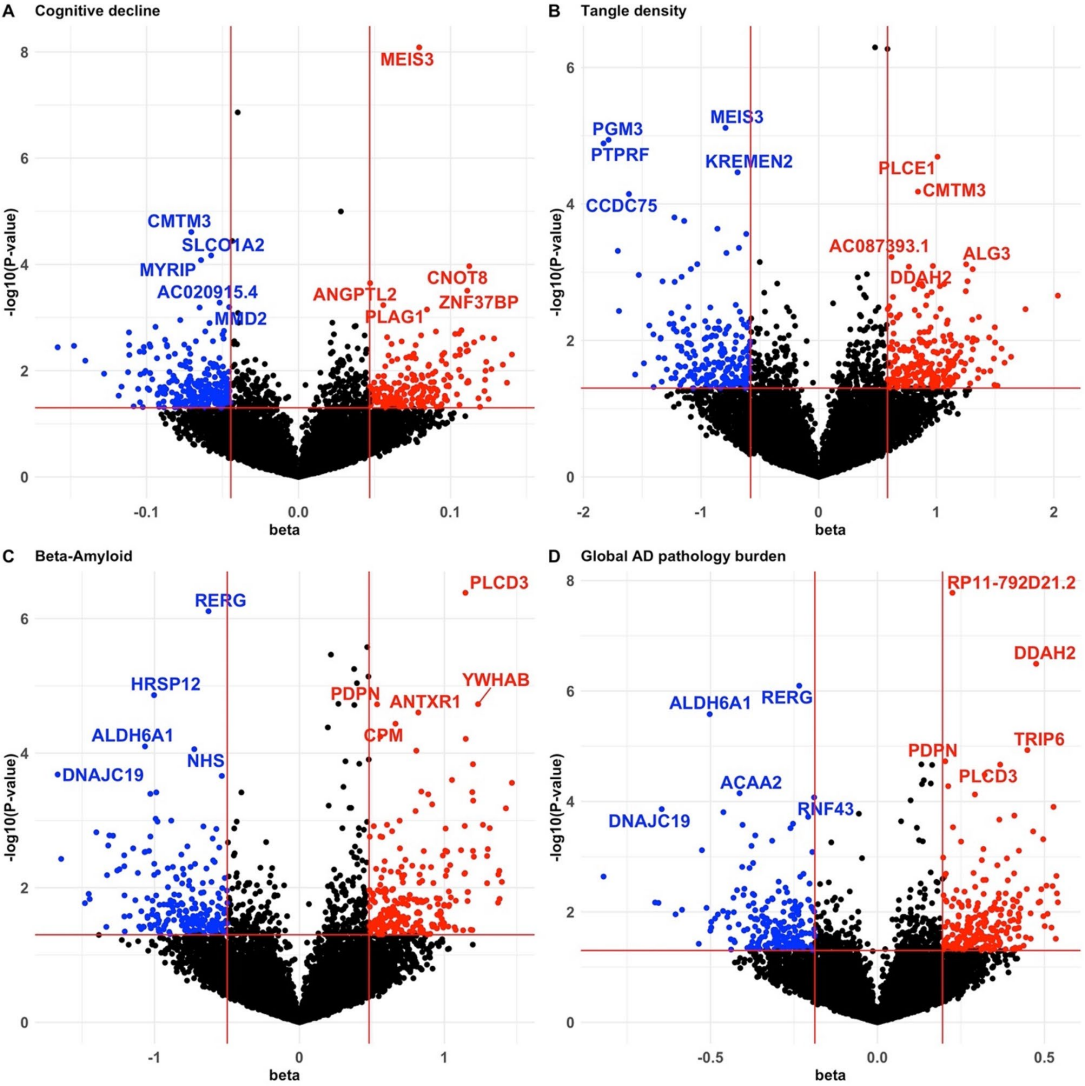
Volcano plots of DGE results by LMM results by LMM with the discovery RNA-Seq data of DLPFC tissue of cognitive decline (**A**), tangle density (**B**), $\beta$-amyloid (**C**), and global AD pathology burden (D). Genes with effect size beta > 0.05 or <  − 0.05 (vertical red lines) and *p*-values < 0.05 (horizontal red line) were colored. Blue points were down regulated genes and red points were up regulated genes. Top five significant up and down regulated genes were labeled.

**Figure 3:**
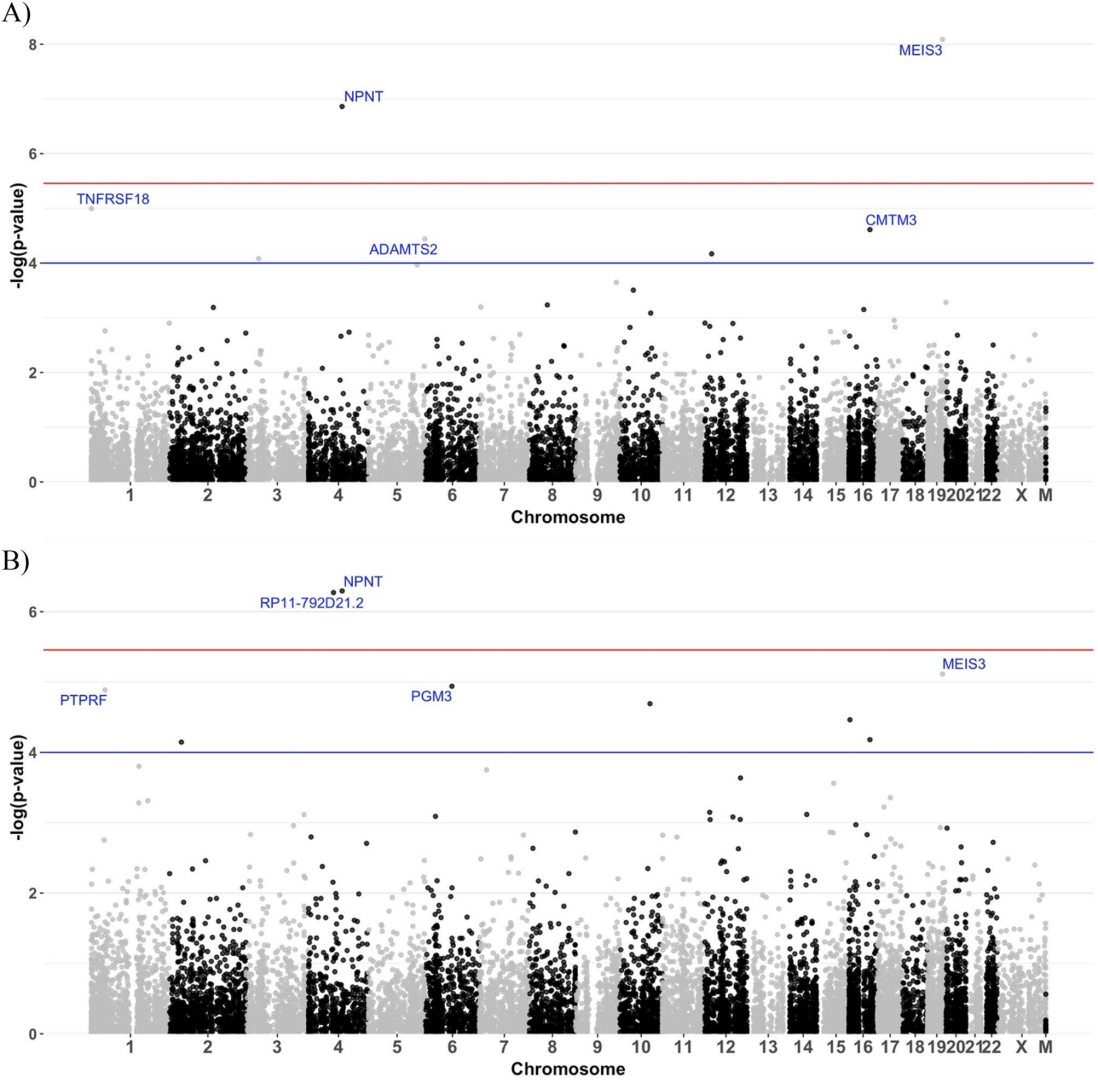
Manhattan plots of DGE p-values by LMM with the discovery RNA-Seq data of DLPFC tissue of cognitive decline (**A**) and tangle density (**B**). Top five significant DGEs were labeled. Red line indicates the significant threshold 3.49 × 10^–6^ with Bonferroni correction and blue line indicates *p*-value = 0.0001.

**Figure 4:**
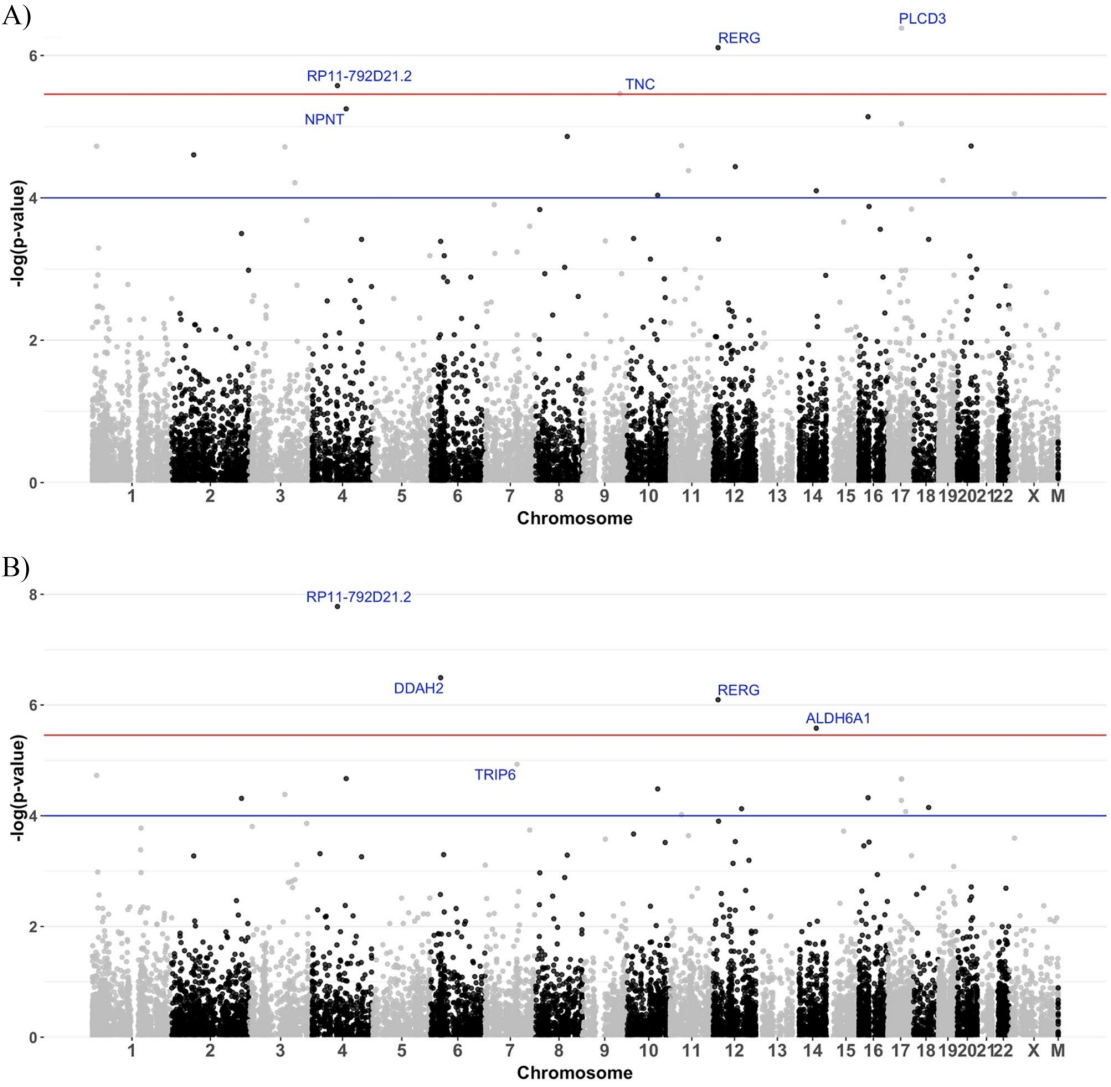
Manhattan plots of DGE p-values by LMM with the discovery RNA-Seq data of DLPFC tissue of $\beta$-amyloid (**A**) and global AD pathology burden (**B**). Top five significant DGEs were labeled. Red line indicates the significant threshold 3.49 × 10^–6^ with Bonferroni correction and blue line indicates *p*-value = 0.0001.

We identified a total of 37 potential statistically significant genes with differential expression (*p*-values < 0.0001 for at least one trait) by the LMM-based test method, including 2 for cognitive decline, 4 for $\beta$-amyloid associated genes, 2 for tangle density, and 4 for global AD pathology burden, with p-values less than the Bonferroni corrected significance threshold of 3.49 × 10^–6^ (Table 2; Figs. 2, 3, 4).

**Table 2:** LMM P-values of 37 potential DGEs (*p*-values < 0.0001) identified by LMM using the discovery ROS/MAP RNA-Seq data of DLPFC, for at least one trait of the cognitive decline and three AD pathologies.

Gene name	CHR	Cognitive decline	$\beta$-Amyloid	Tangle density	Global AD pathology
*PDPN*	1	1.59 × 10^–2^	1.88 × 10^–5^*	2.18 × 10^–2^	1.87 × 10^–5^*
*PTPRF*	1	1.74 × 10^–3^	1.12 × 10^–1^	1.29 × 10^–5^*	2.33 × 10^–2^
*TNFRSF18*	1	1.01 × 10^–5^*	6.60 × 10^–3^	1.17 × 10^–1^	4.23 × 10^–2^
*CCDC75*	2	3.33 × 10^–2^	9.37 × 10^–1^	7.15 × 10^–5^*	1.42 × 10^–1^
*ANTXR1*	2	5.08 × 10^–1^	2.49 × 10^–5^*	1.19 × 10^–1^	5.33 × 10^–4^
*SLC11A1*	2	2.19 × 10^–1^	3.17 × 10^–4^	6.47 × 10^–2^	4.85 × 10^–5^*
*MYRIP*	3	8.30 × 10^–5^*	3.22 × 10^–1^	2.31 × 10^–1^	1.41 × 10^–1^
*CCDC80*	3	5.85 × 10^–1^	1.93 × 10^–5^*	1.06 × 10^–1^	4.13 × 10^–5^*
*C3orf58*	3	3.36 × 10^–1^	6.13 × 10^–5^*	1.48 × 10^–1^	1.43 × 10^–3^
*RP11-792D21.2*^*a*^	4	5.45 × 10^–2^	2.65 × 10^–6^*	5.35 × 10^–7^*	1.66 × 10^–8^*
*NPNT*^*a*^	4	1.38 × 10^–7^*	5.62 × 10^–6^*	5.07 × 10^–7^*	2.13 × 10^–5^*
*ADAMTS2*	5	3.62 × 10^–5^*	6.51 × 10^–4^	6.87 × 10^–3^	3.06 × 10^–3^
*DDAH2*^*a*^	6	7.10 × 10^–1^	4.09 × 10^–4^	8.11 × 10^–4^	3.21 × 10^–7^*
*PGM3*	6	4.65 × 10^–2^	7.32 × 10^–2^	1.15 × 10^–5^*	8.34 × 10^–3^
*TRIP6*	7	6.79 × 10^–2^	5.76 × 10^–4^	3.05 × 10^–3^	1.17 × 10^–5^*
*HRSP12*	8	7.81 × 10^–2^	1.37 × 10^–5^*	8.12 × 10^–2^	5.15 × 10^–4^
*TNC*^*a*^	9	3.82 × 10^–1^	3.43 × 10^–6^*	4.42 × 10^–1^	1.38 × 10^–2^
*PLCE1*	10	5.76 × 10^–3^	9.20 × 10^–5^*	2.03 × 10^–5^*	3.28 × 10^–5^*
*CD44*	11	2.75 × 10^–1^	1.85 × 10^–5^*	3.47 × 10^–2^	9.56 × 10^–5^*
*APLNR*	11	7.49 × 10^–1^	4.14 × 10^–5^*	3.35 × 10^–2^	2.29 × 10^–4^
*RERG*^*a*^	12	1.44 × 10^–3^	7.77 × 10^–7^*	7.10 × 10^–4^	8.03 × 10^–7^*
*SLCO1A2*	12	6.78 × 10^–5^*	2.81 × 10^–1^	9.23 × 10^–2^	1.12 × 10^–1^
*CPM*	12	6.34 × 10^–2^	3.65 × 10^–5^*	1.21 × 10^–2^	2.93 × 10^–4^
*KITLG*	12	1.27 × 10^–3^	4.09 × 10^–2^	8.29 × 10^–4^	7.47 × 10^–5^*
*ALDH6A1*^*a*^	14	4.93 × 10^–1^	7.95 × 10^–5^*	7.63 × 10^–4^	2.63 × 10^–6^*
*KREMEN2*	16	2.97 × 10^–2^	1.80 × 10^–1^	3.44 × 10^–5^*	8.52 × 10^–2^
*APOBR*	16	5.73 × 10^–2^	7.26 × 10^–6^*	1.44 × 10^–2^	4.73 × 10^–5^*
*CMTM3*	16	2.45 × 10^–5^*	1.53 × 10^–2^	6.60 × 10^–5^*	2.48 × 10^–2^
*HIGD1B*	17	1.60 × 10^–1^	1.05 × 10^–3^	1.09 × 10^–2^	5.29 × 10^–5^*
*GFAP*	17	4.13 × 10^–2^	9.11 × 10^–6^*	1.70 × 10^–3^	2.18 × 10^–5^*
*PLCD3*^*a*^	17	5.40 × 10^–1^	4.14 × 10^–7^*	1.14 × 10^–1^	2.15 × 10^–5^*
*RNF43*	17	1.48 × 10^–3^	1.04 × 10^–3^	1.99 × 10^–3^	8.44 × 10^–5^*
*ACAA2*	18	1.80 × 10^–1^	3.83 × 10^–4^	2.61 × 10^–2^	7.10 × 10^–5^*
*PODNL1*	19	8.34 × 10^–1^	5.67 × 10^–5^*	9.29 × 10^–1^	3.86 × 10^–3^
*MEIS3*^*a*^	19	8.21 × 10^–9^*	2.53 × 10^–2^	7.67 × 10^–6^*	8.24 × 10^–4^
*YWHAB*	20	2.57 × 10^–1^	1.87 × 10^–5^*	7.53 × 10^–1^	7.75 × 10^–3^
*NHS*	23	1.34 × 10^–1^	8.74 × 10^–5^*	8.47 × 10^–2^	2.53 × 10^–4^

^a^Significant DGEs with Bonferroni correction (*p*-value < 3.49 × 10^–6^).

*Indicating the corresponding trait (columns 3–6) for which the potential DGE was identified (P-values < 0.0001).

Importantly, many of the potential significant genes associated with cognitive decline and AD-NC traits identified by the LMM method have been reported in prior studies. This confluence of findings bolsters the credibility of our outcomes. Notably, for example, gene *MEIS3* (P-value = 1.16 × 10^–8^ for cognitive decline) and gene *NPNT* (*p*-value = 1.49 × 10^–6^ for tangle density) have previously identified as differentially expressed genes in the context of AD in earlier studies^27,28^. Likewise, Gene *DDAH2* (*p*-value = 4.18 × 10^–7^ for global AD pathology burden) has been linked to increased levels of oxidative stress in AD brains^29^. Furthermore, mutations in the *ALDH* family genes such as *ALDH6A1* (*p*-value = 8.36 × 10^–7^ for global AD pathology burden) were identified as significant risk variants for AD^30^. Additionally, *PLCD3* (*p*-value = 7.64 × 10^–7^ for β-amyloid) is a notable protein that is cross-correlated with β-amyloid and Tau proteins in AD brains^31^. These results indicated the effectiveness of LMM based test for conducting DGE analyses of quantitative traits with well calibrated false positive rates. These intriguing results further underscore the efficacy of the LMM-based approach in facilitating DGE analyses involving quantitative traits, while concurrently maintaining well-calibrated false positive rates.

In summary, 8 of these 37 genes had significant LMM P-values with Bonferroni correction, and 5 of these (*NPNT, ALDH6A1, DDAH2, PLCD3, MEIS3*) were also reported in previous studies^27,28,30^. Interestingly, both *NPNT* and *MEIS3* showed significant differential expression in cognitive decline and tangle density. We also found that 3 of these 37 genes had significant differential expression in cognitive decline and at least one of the AD pathology traits, suggesting a shared gene regulatory mechanism between cognition and AD pathology.

### DGE in the replication RNA-Seq datasets

We applied the LMM, standard linear regression, robust regression, and Voom methods to conduct DGE analyses of the same cognitive decline and AD-NC traits, using additional replication RNA-Seq data of DLPFC (n = 588), SMA (n = 234), spinal cord (n = 232), and muscle (n = 268) tissues (Supplemental Tables 1–4). Confounding covariates such as sex, age, and postmortem interval were adjusted for in all analyses. Study group (ROS or MAP) was only adjusted in DLPFC datasets as participants of SMA, spinal cord, and muscle tissues are all from MAP. QQ-plots (Supplemental Figs. 1–8) still showed that LMM-based test results with these validation datasets were better calibrated than those obtained by standard linear regression, robust regression, and Voom, especially for studying the validation data of DLPFC (Supplemental Figs. 1–3). Thus, we only present the validation results obtained by LMM method here.

With validation RNA-Seq data of DLPFC, we replicated 10 of these 37 potential significant genes identified in the discovery analyses with validation *p*-values<$1.35\times {10}^{-3}$ for either cognitive decline or at least one of the AD-NC traits (Table 3). For example, *PLCD3* differentially expressed for $\beta$-Amyloid and global AD pathology was replicated with P-value = 5.42 × 10^–5^ for global AD pathology; *TRIP6* differentially expressed for global AD pathology was replicated with *p*-value = 6.34 × 10^–4^ for global AD pathology; *PLCE1* differentially expressed for $\beta$-Amyloid, tangle density, and global AD pathology was replicated with *p*-value = 4.51 × 10^–4^ for global AD pathology.

**Table 3:** LMM P-values of 10 replicated DGEs (*p*-value < 0.5/37 = 0.00135) using the validation ROS/MAP RNA-Seq data of DLPFC.

Gene name	CHR	Cognitive decline	$\beta$-Amyloid	Tangle density	Global AD pathology
*PTPRF*	1	2.74 × 10^–1^	1.62 × 10^–2^	1.68 × 10^–2^	1.25 × 10^–3^*
*NPNT*^*a*^	4	3.82 × 10^–6^*	1.22 × 10^–4^*	1.05 × 10^–10^*	3.84 × 10^–10^*
*ADAMTS2*^*a*^	5	7.20 × 10^–5^*	4.33 × 10^–1^	3.14 × 10^–2^	2.14 × 10^–1^
*PGM3*	6	3.89 × 10^–2^	9.08 × 10^–4^*	3.50 × 10^–3^	3.70 × 10^–3^
*TRIP6*	7	6.28 × 10^–2^	6.79 × 10^–2^	6.92 × 10^–6^*	6.34 × 10^–4^*
*PLCE1*	10	4.02 × 10^–1^	2.55 × 10^–3^	2.66 × 10^–3^	4.51 × 10^–4^*
*CD44*	11	1.22 × 10^–2^	9.63 × 10^–1^	4.47 × 10^–4^*	7.39 × 10^–2^
*RERG*^*a*^	12	7.21 × 10^–3^	9.08 × 10^–5^*	1.08 × 10^–7^*	5.45 × 10^–7^*
*PLCD3*	17	3.70 × 10^–1^	1.13 × 10^–3^*	4.05 × 10^–3^	5.42 × 10^–5^*
*MEIS3*	19	2.31 × 10^–5^*	5.58 × 10^–2^	5.18 × 10^–4^*	5.92 × 10^–4^*

^a^Significant DGEs with Bonferroni correction (*p*-value < 2.89 × 10^–6^).

*Indicating the corresponding trait (columns 3–6) for which the potential DGE was replicated (*p*-value < $1.35\times {10}^{-3}$).

Since the validation RNA-Seq datasets of the motor function related tissues (SMA, spinal cord, muscle) have sample sizes of only ~ 100 (Supplemental Table 3), we used a more liberal p-value threshold (nominal *p*-value < 0.05 for either cognitive decline or at least one of the AD-NC traits) to identify replicated genes. As a result, for the replicated differentially expressed genes in the CNS tissues, we found 8 in SMA with 5 overlapped in DLPFC, 2 in spinal cord, 3 in muscle with one overlapped in spinal cord, and one overlapped in SMA (Table 4). For example, *ALDH6A1* differentially expressed for global AD pathology in the discovery data was replicated with P-value = 0.008 for cognitive decline in SMA and muscle. Additionally, *HRSP12*, a differentially expressed gene related to cognitive decline in the discovery analyses was replicated with significant *p*-values < 0.0001 in SMA. Interestingly, several differentially expressed genes including *ADAMTS2* for cognitive decline, *NPNT* for all four traits, *RERG* for $\beta$-Amyloid and global AD pathology, as well as *MEIS3* for cognitive decline and tangle density that were identified in the discovery data were replicated in the validation datasets of DLPFC and CNS tissues. It is noteworthy that replicated gene *ADAMTS2* in both DLPFC and SMA tissues was suggested to be a therapeutic target for AD^32^, while replicated gene *HRSP12* in SMA is also known by its alias *RIDA,* which was found as a GWAS risk loci for blood protein levels by previous studies^33^.

**Table 4:** LMM P-values of 11 replicated DGEs (*p*-value < 0.05) using the validation ROS/MAP RNA-Seq data of SMA, spinal cord and muscle tissues.

Tissue	Gene name	CHR	Cognitive decline	$\beta$-Amyloid	Tangle density	Global AD pathology
SMA	*PTPRF*	1	0.201	0.015*	0.589	0.122
	*NPNT*	4	0.121	0.463	0.011*	0.147
	*ADAMTS2*^*a*^	5	0.001*	0.014*	3.0 × 10^–5^*	0.001*
	*HRSP12*^*a*^	8	4.4 × 10^–5^*	0.010*	0.028*	0.007*
	*PLCE1*	10	0.056	0.935	0.016*	0.106
	*RERG*	12	0.05	0.141	0.048*	0.122
	*CPM*	12	0.3	0.165	0.003*	0.011*
	*ALDH6A1*	14	0.008*	0.196	0.05	0.049*
Spinal cord	*APOBR*	6	0.7	0.153	0.135	0.039*
	*APLNR*	11	0.17	0.035*	0.114	0.06
Muscle	C3orf58	3	0.033*	0.389	0.222	0.478
	*APLNR*	11	0.22	0.015*	0.505	0.127
	*ALDH6A1*	14	0.043*	0.352	0.204	0.121

^a^Significantly replicated DGEs with *p*-value < 0.001.

*Indicating the corresponding trait (columns 4–7) for which the potential DGE was replicated (*p*-value < 0.05).

To further illustrate the reason why differentially expressed genes in the DLPFC tissue could be replicated in the SMA, spinal cord, and muscle tissues, we created correlation heatmaps of the gene expression levels of these validated genes. For each trait, we sorted samples based on their trait values and divided sorted samples into ten equal parts (i.e., decile). For each replicated gene, we calculated the average gene expression level of the discovery DLPFC and replication tissues, for samples in each decile of the discovery and replicated traits. Then we calculated the correlations between these two vectors of average gene expression. Heatmaps of these correlations (Supplemental Figs. 9,10) show that most correlations are > 0.15, demonstrating that these replicated differentially expressed genes are not tissue specific, but likely to be shared across these motor function related tissues. For example, differentially expressed genes *ADAMTS2* and *HRSP12* that were replicated with all four traits in SMA all have gene expression correlations > 0.15.

In conclusion, our analysis revealed that all 5 differentially expressed genes (*NPNT, ALDH6A1, RERG, PLCD3, MEIS3*) with Bonferroni corrected significant p-values in the discovery data were replicated in the validation data of DLPFC tissue. Furthermore, we validated a total of 17 unique genes across the validation RNA-Seq data of DLPFC and three motor function related tissues, including 10 in DLPFC, 8 in SMA, 2 in spinal cord, and 3 in muscle. The identification of shared differentially expressed genes in two different tissue types, such as DLPFC and SMA, DLPFC and non-neural muscle, as well as spinal cord and non-neural muscle outside the brain, suggests a possible shared molecular mechanism between motor and cognition functions.

### Pathway enrichment analysis

**Figure 5:**
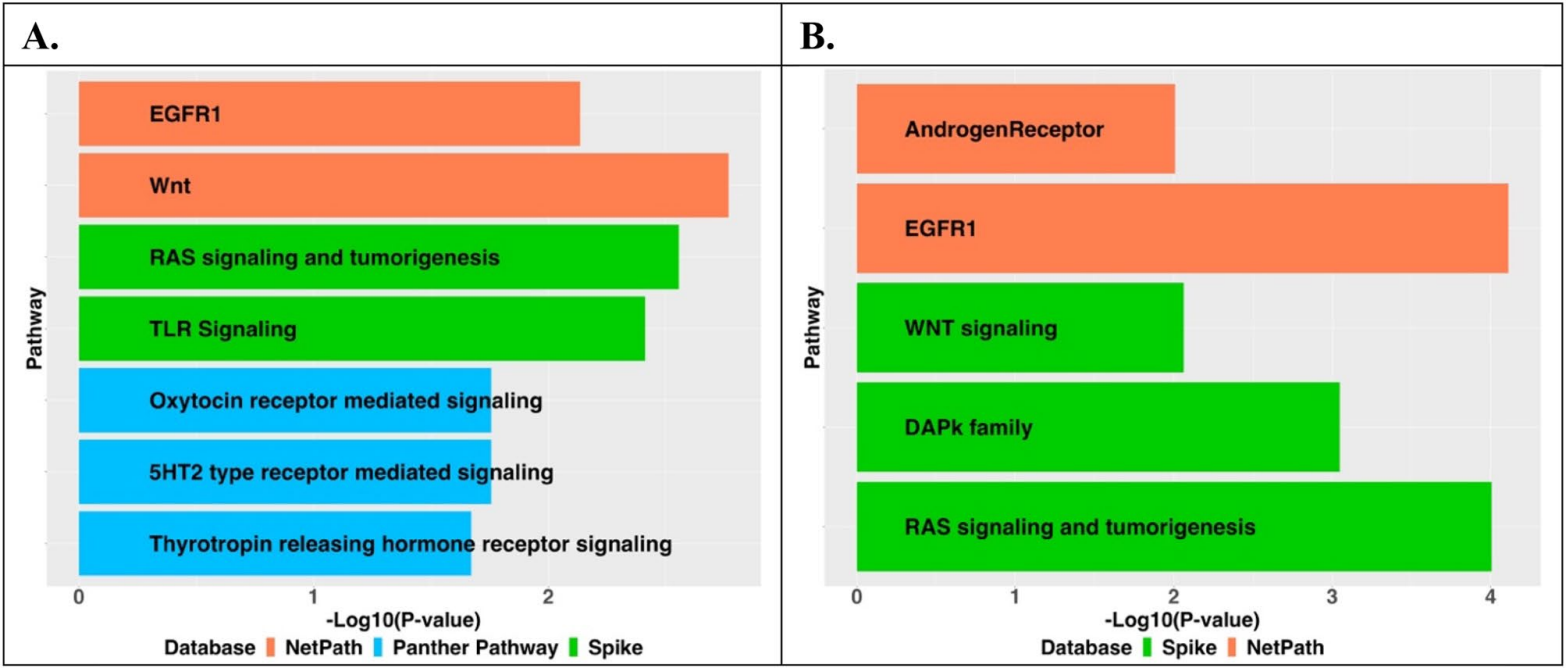
Significant pathways with FDR < 0.05 that are enriched with top 100 differentially expressed genes of cognitive decline (**A**) and tangle density (**B**) with the discovery RNA-Seq data of DLPFC tissue.

To illustrate the underlying pathways and biological functions of our identified differentially expressed genes of all 4 AD traits. We selected top 100 significantly differentially expressed genes identified by using the discovery RNA-Seq data of DLPFC tissue for each of the 4 traits to conduct pathway enrichment analyses by pathDIP^34^. Databases of NetPath^35^, Panther Pathway^36^, and Spike^37^ were used in the enrichment analyses. Significant enrichment in several biological pathways were identified with the top 100 significantly differentially expressed genes of cognitive decline and tangle density (Fig. 5). These significant pathways were reported by previous studies to be relevant with AD.

For example, for the pathways significantly enriched with top 100 differentially expressed genes of cognitive decline (Fig. 5A), the Thyrotropin releasing hormone (TRH) receptor signaling pathway (FDR = 3.54 × 10^–2^) has been associated with aging and neurodegenerative diseases, such as Alzheimer's disease and Parkinson's disease^38^. Similarly, the 5HT2 type receptor mediated signaling pathway (FDR = 4.37 × 10^–2^) could influence the behavioral and psychological symptoms of dementia (BPSD) in Alzheimer's disease (AD)^39^. The Oxytocin receptor medicated signaling pathway (FDR = 4.37 × 10^–2^) was suggested to be a novel protective target for vascular dementia and mixed dementias^40^. The toll-like receptor (TLR) signaling pathway (FDR = 1.42 × 10^–2^) may be involved in clearance of amyloid β-protein (Aβ) in the brain making it a potential therapeutic target for AD^41^. The Renin-Anigotensin System (RAS) and tumorigenesis pathway (FDR = 1.52 × 10^–2^) is known to play a key role in interacting with pathophysiological mechanisms of AD^42^. Several evidences suggest that enhancing Wnt pathway (FDR = 1.88 × 10^–2^) can boost synaptic function during aging, and ameliorate synaptic pathology in AD which could be novel therapeutic for restoration in the brain^43^. The Epidermal growth factor receptor (EGFR1) pathway (FDR = 2.01 × 10^–2^), a preferred target for treating memory loss induced by amyloid-beta (Aβ)^44^, is also enriched in β-amyloid.

Also, for the pathways significantly enriched with top 100 differentially expressed genes of tangle density (Fig. 5B), Death-Associated Protein Kinase 1 in DAPk family (FDR = 5.98 × 10^–3^) that plays a critical role in deregulation in AD thus manipulating DAPK1 activity and/or expression could be a promising drug target in AD^45^. The additional protective mechanism of AndrogenReceptor (FDR = 3.92 × 10^–2^) might enhance neural health and deter the progression of AD^46^.

## Discussion

Most existing DGE analysis methods^6–9,11^ are developed for dichotomous traits with small sample sizes. However, with increased sample sizes in RNA-Seq datasets, there is a huge demand for methods for studying quantitative traits in population-based RNA-Seq studies. As shown by the MACAU^11^ method paper, incorporating a mixed term into DGE analysis can help to control for false positive rates in RNA-Seq studies. In this study, we develop an analytic pipeline for implementing the GEMMA tool^22^, enabling the DGE analysis of quantitative traits by LMM, and apply to real ROS/MAP RNA-Seq datasets of DLPFC, SMA, spinal cord, and muscle tissues for studying continuous cognitive decline and AD-NC traits. The pipeline is freely available from https://github.com/tangjiji199645/LMM_DGE_Pipeline.

Our application studies found that DGE analyses results obtained by LMM-based tests were all well calibrated for false positive rate, especially in our discovery RNA-Seq data of DLPFC, while the DGE results obtained by the alternative standard linear regression, robust regression, and Voom methods all have inflated false positive rates. A list of 37 potential differentially expressed genes were identified by LMM in the discovery data, and 17 of these were replicated in the additional RNA-seq data of DLPFC, SMA, spinal cord, and muscle tissues. AD relevant biological pathways were also found to be enriched with top differentially expressed genes of cognitive decline and tangle density.

However, the LMM-based test still has several limitations for studying RNA-Seq data. First, the LMM assumes normal distributions for both the response variable and covariates, while the raw RNA-Seq data are read counts per gene. Even after log2 transformation for the raw RNA-Seq read counts, the transformed quantitative gene expression level per gene may not be normally distributed^47^, which could lead to a biased estimation of effect sizes. One might apply both the MACAU (mixed Poisson model-based test that is suitable for modeling RNA-Seq read counts) and our LMM analytic pipeline for DGE analysis and examine the results by QQ-plots. Second, the effect of the gene expression on the quantitative trait of interest may be heterogeneous, with effect sizes varying across the quantiles of the quantitative trait. The LMM-based method only tests the association between gene expression and quantitative trait of interest in expectation, ignoring the possible heterogeneous effects on different quantiles of the quantitative trait^48^. Therefore, further studies are needed to develop a DGE method based on quantile regression with a mixed effect term to account for the possible heterogeneous effect of gene expression across all the quantiles of the quantitative trait of interest, while controlling for false positive rates.

Overall, we provide a useful LMM pipeline for conducting DGE analysis with quantitative traits and large sample sizes, which is shown well calibrating for false positive rates in real studies. Our real application studies not only demonstrated the effectiveness of the LMM approach for DGE analysis, but also identified a list of differentially expressed genes for cognitive decline and AD-NC traits in DLPFC that were validated in DLPFC, SMA, spinal cord, and muscle tissues. Our findings have important implications for understanding the underlying biological mechanisms of the continuous AD traits of cognitive decline and neuropathologic changes, and may provide insights into the development of new therapeutic approaches for AD.

## Methods

### RNA-Seq data normalization

Preprocessing and normalization of raw read counts is a critical step in DGE analysis. Generally, samples with total mapped reads < 10 million are suggested to be excluded, and genes with expression levels < 0.1 transcript per million (TPM) in > 20% samples are also suggested to be excluded. We use DESeq2^6^ to normalize raw RNA-Seq data.

Let ${{\varvec{K}}}_{{\varvec{i}}{\varvec{j}}}$ denote the read count for gene ***j*** and sample ***i***, following a negative binomial distribution with mean ${{\varvec{\mu}}}_{{\varvec{i}}{\varvec{j}}}$ and dispersion ${\boldsymbol{\alpha }}_{{\varvec{j}}}$ given by the following formulas:

1 $${\varvec{K}}_{{{\varvec{ij}}}} \sim {\varvec{NB}}\left( {{\varvec{\mu}}_{{{\varvec{ij}}}} ,{\varvec{\alpha}}_{{\varvec{j}}} } \right),$$

2 $${\varvec{\mu}}_{{{\varvec{ij}}}} = {\varvec{s}}_{{{\varvec{ij}}}} {\varvec{x}}_{{{\varvec{ij}}}} \user2{ }\user2{.}$$

The normalization factor ${{\varvec{s}}}_{{\varvec{i}}{\varvec{j}}}$ is assumed to be shared per sample, ${{\varvec{s}}}_{{\varvec{i}}{\varvec{j}}}={{\varvec{s}}}_{{\varvec{i}}}$, and ${{\varvec{s}}}_{{\varvec{i}}}$ is estimated by the median (across all genes) of the ratios of raw read count per gene and its corresponding geometric mean ${{\varvec{K}}}_{{\varvec{i}}}^{{\varvec{R}}}$ (across all samples) as in the following formula^49,50^:

3 $${\varvec{s}}_{{\varvec{i}}} = {\varvec{median}}_{{{\varvec{j}}:{\varvec{K}}_{{\varvec{j}}}^{{\varvec{R}}} \ne 0}} \frac{{{\varvec{K}}_{{{\varvec{ij}}}} }}{{{\varvec{K}}_{{\varvec{j}}}^{{\varvec{R}}} }}\;with\;{\varvec{K}}_{{\varvec{i}}}^{{\varvec{R}}} = \left( {\mathop \prod \limits_{{{\varvec{i}} = 1}}^{{\varvec{m}}} {\varvec{K}}_{{{\varvec{ij}}}} } \right)^{{1/{\varvec{m}}}} .$$

Normalized read counts ${{\varvec{x}}}_{{\varvec{i}}{\varvec{j}}}$ are given by $\frac{{K}_{ij}}{{s}_{ij}}$ and then log2 transformed with an offset of 1 and taken as the test variable in the LMM, standard linear regression, robust regression, and Voom methods.

### Standard linear regression model for DGE analysis of quantitative traits

As proposed by previous study^21^, testing if gene $j$ with expression levels ${{\varvec{X}}}_{{\varvec{j}}}$ **(**normalized and log2 transformed**)** is differentially expressed with respect to a quantitative trait ${{\varvec{Y}}}_{{\varvec{n}}\times 1}$ can be done based on the following standard linear regression :

4 $${\varvec{Y}} = \user2{ W\alpha } + \user2{ X}_{{\varvec{j}}} \beta_{j} \user2{ } + \user2{ \varepsilon },\user2{ }$$

5 $$\user2{\varepsilon }\sim \user2{ MVN}\left( {0,{\varvec{I}}_{{\varvec{n}}} \sigma^{2} } \right),$$

where ***n*** is the number of test samples; ${\varvec{W}}$ is a ${\varvec{n}}\times {\varvec{c}}$ covariate matrix; $\boldsymbol{\alpha }$ is a ${\varvec{c}}\times 1$ vector of covariate effects including the intercept; ${{\varvec{\beta}}}_{{\varvec{j}}}$ is the effect size of gene ***j***; and ${\varvec{\epsilon}}$ denotes the error term following a Multivariate Normal distribution (MVN). The DGE analysis is to test the null hypothesis of ${H}_{0}: {\beta }_{j}=0\boldsymbol{ }vs. {H}_{a}:{\beta }_{j}\ne 0$, which can be conducted by using the Wald test statistic,

$$\frac{\widehat{{\beta }_{j}}}{se(\widehat{{\beta }_{j}})}\sim N(0, 1) \,{\text u \text n\text d\text e \text r}\, {H}_{0},$$

with the maximizing likelihood estimator $\widehat{{\beta }_{j}}$ and its standard error $se(\widehat{{\beta }_{j}})$.

### Robust regression for DGE analysis of quantitative traits

Robust regression^21^ assumes the same model as the standard linear regression model (4), yet it furnishes robust coefficient estimates when the test samples contain influential outliers that could heavily impact standard linear regression estimates. Different from the standard linear regression method where each sample contributes equally to the ordinary least squared estimation of regression coefficients, robust regression incorporates Huber’s M-estimation^51^ that is obtained by minimizing the following objection function through a numerical method called iteratively reweighted least squares (IRLS):

$$\mathop \sum \limits_{i = 1}^{n} \rho \left( {Y_{i} - W_{i} \alpha - X_{i,j} \beta_{j} } \right),\; with\; \rho \left( e \right) = \left\{ {\begin{array}{*{20}l} {\frac{1}{2}e^{2} } \hfill & { if \left| e \right| < k} \hfill \\ {k\left| e \right| - \frac{1}{2}k^{2} } \hfill & {if \left| e \right| \ge k} \hfill \\ \end{array} } \right., \;w\left( e \right) = \left\{ {\begin{array}{*{20}c} 1 & {if \left| e \right| \le k} \\ {\frac{k}{\left| e \right|}} & { if \left| e \right| \ge k} \\ \end{array} } \right. ,$$

where $k$ is the tuning constant (often taken as 1.345 $\sigma$); $\rho (e)$ is Huber’s objective function; and $w(e)$ is Huber’s weighted function. The weighted least squares estimate with sample weights given by $w({Y}_{i}-{W}_{i}\widehat{\alpha }-{X}_{i,j}\widehat{{\beta }_{j}})$ will be iteratively calculated given coefficient estimates from last iteration, until a stopping criterion is met.

As described in the previous study that proposed using the robust regression for DGE analysis^21^, the R packages of “rlm” and “sfsmisc” can be used to conduct the statistical test of ${H}_{0}: {\beta }_{j}=0\boldsymbol{ }vs. {H}_{a}:{\beta }_{j}\ne 0$. The robust regression method is expected to provide more reliable estimates of ${\beta }_{j}$ when outliers are present in the test samples.

### Voom for DGE analysis

Since the Voom method^10^ is developed for detecting differentially expressed genes between two or more conditions, the quantitative trait ${{\varvec{Y}}}_{{\varvec{n}}\times 1}$ needs to be dichotomized for testing if gene $j$ with expression levels ${{\varvec{X}}}_{{\varvec{j}}}$ **(**normalized and log2 transformed read counts**)** is differentially expressed. Generally, the quantitative trait is dichotomized to ${{\varvec{Y}}}_{{\varvec{n}}\times 1}^{{\varvec{d}}}$ by taking the median as a cut-off. That is, if ${{\varvec{Y}}}_{{\varvec{i}}}^{{\varvec{d}}}$ is greater than the median, it is assigned a value of 1, otherwise ${{\varvec{Y}}}_{{\varvec{i}}}^{{\varvec{d}}}$ is assigned a value of 0. The Voom method assumes the following model:

$${\varvec{E}}\left( {{\varvec{X}}_{{{\varvec{ij}}}} } \right) = \user2{ W}_{{\varvec{i}}} \user2{\alpha } + \user2{ Y}_{{\varvec{i}}}^{{\varvec{d}}} {\varvec{\beta}}_{{\varvec{j}}} \user2{ }$$

where ${\varvec{W}}$ is a ${\varvec{n}}\times {\varvec{c}}$ matrix of confounding covariates; $\boldsymbol{\alpha }$ is a ${\varvec{c}}\times 1$ vector of covariate effects including an intercept term; ${\beta }_{j}$ is coefficient of gene ***j*** representing log2-fold-changes between two conditions of the dichotomous trait. The same hypothesis with ${H}_{0}: {\beta }_{j}=0\boldsymbol{ }\; vs. {H}_{a}:{\beta }_{j}\ne 0$ is tested by Voom. Different from the ordinary least squared estimates based on (10), the Voom method robustly estimates the mean–variance relationship of the log2 transformed read counts, generates a precision weight for each sample, and enters these into the limma empirical Bayes analysis pipeline^10^.

### LMM by GEMMA

To test if gene $j$ with expression levels ${{\varvec{X}}}_{{\varvec{j}}}$** (**normalized and log2 transformed read counts**)** is differentially expressed with respect to a quantitative trait ${{\varvec{Y}}}_{{\varvec{n}}\times 1}$ with $n$ samples in DGE analysis, the following LMM is assumed:

6 $${\varvec{Y}} = \user2{ W\alpha } + \user2{ X}_{{\varvec{j}}} \beta_{j} \user2{ } + \user2{ Zu } + \user2{ \varepsilon },\user2{ }j = 1, ..., p$$

7 $${\varvec{u}}\sim {\varvec{MVN}}\left( {0,\user2{\gamma \tau }^{ - 1} {\varvec{M}}} \right),$$

8 $${\varvec{\varepsilon}}\sim {\varvec{MVN}}\left( {0,{\varvec{\tau}}^{ - 1} {\varvec{I}}_{{\varvec{n}}} } \right),$$

where ${\varvec{W}}$ is a ${\varvec{n}}\times {\varvec{c}}$ matrix of confounding covariates; $\boldsymbol{\alpha }$ is a ${\varvec{c}}\times 1$ vector of covariate effects including an intercept term; ${\beta }_{j}$ is the effect size of gene ***j***; ***Z*** is a ${\varvec{n}}\times {\varvec{n}}$ loading matrix which is taken as an identify matrix for DGE analysis; ${\varvec{\varepsilon}}$ is a ${\varvec{n}}\times 1$ vector of independent errors following a normal distribution with mean 0 and variance ${{\varvec{\tau}}}^{-1}$; ***u*** is a ${\varvec{n}}\times 1$ vector denoting random effects of all samples following a Multivariate Normal distribution with mean **0** and variance–covariance matrix ${\varvec{\gamma}}{{\varvec{\tau}}}^{-1}{\varvec{M}}$; ${\varvec{\gamma}}$ is the ratio of variance components between random effects and errors; ${\varvec{M}}$ is a ${\varvec{n}}\times {\varvec{n}}$ sample-sample correlation matrix with all gene expressions; and ${{\varvec{I}}}_{{\varvec{n}}}$ is an ${\varvec{n}}\times {\varvec{n}}$ identity matrix.

GEMMA tool^22^ is a C++ programed software that can be used to conduct tens of thousands of Wald test for ${H}_{0}:{\beta }_{j}=0 (j=1, \dots , p)$. Under the above LMM, GEMMA efficiently calculates the REstricted Maximum Likelihood (REML) estimates of ${\varvec{\gamma}},\boldsymbol{ }\boldsymbol{ }\boldsymbol{ }{\beta }_{j},$ and the standard error of ${\beta }_{j}$. Although GEMMA is originally developed for genome-wide association study to test the association between a genetic variant and a quantitative trait, it can be applied to DGE if both genetic variant and log2 transformed gene expression follow normal distributions.

We demonstrate the feasibility and effectiveness of conducting DGE analyses using the LMM method through application studies with the ROS/MAP data^17^. Our analyses were conducted in two steps. First, we normalized raw RNA-Seq data using DESeq2^6^. Second, we tested DGE using the LMM method as implemented in the GEMMA tool^22^.

### ROS/MAP data

The Religious Order Study (ROS) and the Rush Memory and Aging Project (MAP) are two prospective community-based harmonized cohort studies of aging, which recruit senior individuals without known dementia at study entry^52^. All ROSMAP participants agree to structured annual clinical testing and autopsy and brain donation upon their death. RNA-Seq data (DLPFC, SMA, spinal cord, and muscle tissues) and AD pathologies were profiled from decedents. Both studies were approved by the Institutional Review Board of Rush University Medical Center, and all participants signed informed and repository consents and an Anatomic Gift Act.

### Alzheimer’s disease clinical and neuropathologic traits

Our study focused on cognitive decline and three AD-NC traits, including *β*-amyloid, tangle density, and global AD pathology burden. The cognitive decline (annual rate of cognitive decline) is the estimated person-specific rate of change in the global cognition variable over all follow-ups generated by a mixed effects model^53,54^. The AD-NC trait of tangle density was quantified using molecularly specific immunohistochemistry. It was profiled as the average PHFtau tangle density within two or more 20 µm sections from eight brain regions—hippocampus, entorhinal cortex, midfrontal cortex, inferior temporal, angular gyrus, calcarine cortex, anterior cingulate cortex, and superior frontal cortex. These two are identified by molecularly specific immunohistochemistry. Trait *β*-Amyloid quantifies the average percent area of cortex occupied by *β*-Amyloid protein in adjacent sections from the same eight brain regions. The global AD pathology burden is a quantitative summary of AD pathology derived from counts of three AD pathologies: neuritic plaques, diffuse plaques, and neurofibrillary tangles with 5 brain regions midfrontal cortex, midtemporal cortex, inferior parietal cortex, entorhinal cortex, and hippocampus (total 15 regional counts). To improve normality, these three quantitative AD pathology traits were transformed by taking the square root. Futher details have been previously reported^55,56^.

### RNA-Seq data of DLPFC, SMA, spinal cord, and muscle tissues

RNA-Seq data were profiled from deceased ROS/MAP participants for DLPFC tissue within the brain (*n* = 1220, *n* = 632 as discovery data Table 1, *n* = 588 as validation data; Supplemental Table 1) and three validation tissues (Supplemental Tables 2–4)––SMA in the brain (*n* = 234), contralateral ventral horn in the lumbar spinal cord (outside the brain, *n* = 232), and non-neural quadriceps muscle ipsilateral to the ventral horn (outside the brain, *n* = 268). The raw RNA-Seq fastq data were first aligned to the reference human genome and then quantified by the number of reads mapped to gene regions. Raw read counts were first normalized and log2 transformed by DESeq2, and then used as the test gene expression covariates in the LMM model. The ROS/MAP RNA-Seq data of DLPFC (*n* = 632) were analyzed as discovery data, while RNA-Seq datasets another 588 DLPFC samples, and samples of SMA, spinal cord, and muscle tissues were analyzed as validation data. Participants are not overlapped between the DLPFC samples and samples of SMA, spinal cord, and muscle tissues, while participants of RNA-Seq data of SMA, spinal cord, and muscle tissues are largely overlapped. Technical details of RNA-Seq data profiling can be found in the Supplemental Text.

### Ethics declarations

The Religious Order Study (ROS) and the Rush Memory and Aging Project (MAP) were approved by the Institutional Review Board of Rush University Medical Center, and all participants signed informed and repository consents and an Anatomic Gift Act. All data analyzed in this study were de-identified which are not considered as human data according to NIH protocols. We confirm that all analytical methods were performed in accordance with the relevant guidelines and regulations.

### Supplementary Information

Supplementary Information.

## Data Availability

All data analyzed in this study are de-identified and available to any qualified investigator with the application through the Rush Alzheimer’s Disease Center Research Resource Sharing Hub, https://www.radc.rush.edu, which has descriptions of the studies and available data. Part of the RNA-Seq data of DLPFC samples are deposited into Synapse, https://doi.org/10.7303/syn3388564. The LMM pipeline for DGE analysis with quantitative traits and large sample sizes is provided at Github, https://github.com/tangjiji199645/LMM_DGE_Pipeline.

## References

[1] Behjati S, Tarpey PS (2013). What is next generation sequencing?. Archiv. Dis. Childhood Educ. Pract. Edn..

[2] Reuter JA, Spacek DV, Snyder MP (2015). High-throughput sequencing technologies. Mol Cell.

[3] Kukurba KR, Montgomery SB (2015). RNA sequencing and analysis. Cold Spring Harbor Protocols.

[4] Costa-Silva J, Domingues D, Lopes FM (2017). RNA-Seq differential expression analysis: An extended review and a software tool. PloS One.

[5] Young MD, Rodríguez-Ezpeleta N, Hackenberg M, Aransay AM (2012). Bioinformatics for High Throughput Sequencing.

[6] Love MI, Huber W, Anders S (2014). Moderated estimation of fold change and dispersion for RNA-seq data with DESeq2. Genome Biol..

[7] Robinson MD, McCarthy DJ, Smyth GK (2010). edgeR: a Bioconductor package for differential expression analysis of digital gene expression data. Bioinformatics.

[8] McCarthy DJ, Chen Y, Smyth GK (2012). Differential expression analysis of multifactor RNA-Seq experiments with respect to biological variation. Nucleic Acids Res..

[9] Ritchie ME (2015). limma powers differential expression analyses for RNA-sequencing and microarray studies. Nucleic Acids Res..

[10] Law CW, Chen Y, Shi W, Smyth GK (2014). voom: precision weights unlock linear model analysis tools for RNA-seq read counts. Genome Biol..

[11] Sun S (2017). Differential expression analysis for RNAseq using Poisson mixed models. Nucleic Acids Res..

[12] Bateman RJ (2012). Clinical and biomarker changes in dominantly inherited Alzheimer's disease. N. Engl. J. Med..

[13] Boyle PA (2019). Attributable risk of Alzheimer's dementia attributed to age-related neuropathologies. Ann. Neurol..

[14] Melikyan ZA (2022). Cognitive resilience to three dementia-related neuropathologies in an oldest-old man: A case report from The 90+ Study. Neurobiol. Aging.

[15] Twine NA, Janitz K, Wilkins MR, Janitz M (2011). Whole transcriptome sequencing reveals gene expression and splicing differences in brain regions affected by Alzheimer's disease. PLoS One.

[16] Consortium G (2020). The GTEx Consortium atlas of genetic regulatory effects across human tissues. Science.

[17] De Jager PL (2018). A multi-omic atlas of the human frontal cortex for aging and Alzheimer’s disease research. Sci. Data.

[18] Fromer M (2016). Gene expression elucidates functional impact of polygenic risk for schizophrenia. Nat. Neurosci..

[19] Consortium, G. T. (2017). Genetic effects on gene expression across human tissues. Nature.

[20] Hoffman GE (2019). CommonMind Consortium provides transcriptomic and epigenomic data for Schizophrenia and Bipolar Disorder. Scientific Data.

[21] Seo M (2016). RNA-seq analysis for detecting quantitative trait-associated genes. Sci. Rep..

[22] Zhou X, Stephens M (2012). Genome-wide efficient mixed-model analysis for association studies. Nat. Genet..

[23] Chen H (2016). Control for population structure and relatedness for binary traits in genetic association studies via logistic mixed models. Am. J. Hum. Genet..

[24] Loh P-R, Kichaev G, Gazal S, Schoech AP, Price AL (2018). Mixed-model association for biobank-scale datasets. Nat. Genet..

[25] Buchman AS, Bennett DA (2011). Loss of motor function in preclinical Alzheimer's disease. Expert Rev. Neurother..

[26] Devlin B, Roeder K (1999). Genomic control for association studies. Biometrics.

[27] Li QS, De Muynck L (2021). Differentially expressed genes in Alzheimer’s disease highlighting the roles of microglia genes including OLR1 and astrocyte gene CDK2AP1. Brain Behav. Immun. Health.

[28] Panitch R (2021). Integrative brain transcriptome analysis links complement component 4 and HSPA2 to the APOE ε2 protective effect in Alzheimer disease. Mol. Psychiatry.

[29] Cioffi F, Adam RHI, Bansal R, Broersen K (2021). A review of oxidative stress products and related genes in early alzheimer's disease. J. Alzheimers Dis.

[30] Vasiliou V, Nebert DW (2005). Analysis and update of the human aldehyde dehydrogenase (ALDH) gene family. Hum Genom..

[31] Hales CM (2016). Changes in the detergent-insoluble brain proteome linked to amyloid and tau in Alzheimer's Disease progression. Proteomics.

[32] Yamakage Y (2019). A disintegrin and metalloproteinase with thrombospondin motifs 2 cleaves and inactivates Reelin in the postnatal cerebral cortex and hippocampus, but not in the cerebellum. Mol. Cell. Neurosci..

[33] Sun BB (2018). Genomic atlas of the human plasma proteome. Nature.

[34] Rahmati S, Abovsky M, Pastrello C, Jurisica I (2017). pathDIP: An annotated resource for known and predicted human gene-pathway associations and pathway enrichment analysis. Nucleic Acids Res..

[35] Kandasamy K (2010). NetPath: A public resource of curated signal transduction pathways. Genome Biol..

[36] Mi H (2019). Protocol Update for large-scale genome and gene function analysis with the PANTHER classification system (v.14.0). Nat. Protocols.

[37] Elkon R (2008). SPIKE—A database, visualization and analysis tool of cellular signaling pathways. BMC Bioinform..

[38] Daimon CM, Chirdon P, Maudsley S, Martin B (2013). The role of Thyrotropin Releasing Hormone in aging and neurodegenerative diseases. Am. J. Alzheimers Dis..

[39] Tang L (2017). The association between 5HT2A T102C and behavioral and psychological symptoms of dementia in alzheimer's disease: A meta-analysis. Biomed. Res. Int..

[40] Counts SE (2020). Therapeutic potential of oxytocin receptor signaling in vascular dementia. Alzheimer's Dementia.

[41] Tahara K (2006). Role of toll-like receptor signalling in Aβ uptake and clearance. Brain.

[42] Ribeiro VT, de Souza LC, Simões ESAC (2020). Renin-angiotensin system and alzheimer's disease pathophysiology: From the potential interactions to therapeutic perspectives. Protein Pept. Lett..

[43] Palomer E, Buechler J, Salinas PC (2019). Wnt signaling deregulation in the aging and alzheimer’s brain. Front. Cell. Neurosci..

[44] Wang L (2012). Epidermal growth factor receptor is a preferred target for treating amyloid-β–induced memory loss. Proc. Natl. Acad. Sci..

[45] Chen D, Zhou XZ, Lee TH (2019). Death-associated protein kinase 1 as a promising drug target in cancer and alzheimer's disease. Recent Pat. Anticancer Drug Discov..

[46] Yao M, Rosario ER, Soper JC, Pike CJ (2022). Androgens regulate tau phosphorylation through phosphatidylinositol 3-kinase-protein kinase B-glycogen synthase kinase 3β signaling. Neuroscience.

[47] Dündar, F., Skrabanek, L. & Zumbo, P. Introduction to differential gene expression analysis using RNA-seq. *Appl. Bioinformatics*, 1–67 (2015).

[48] Song X (2017). QRank: a novel quantile regression tool for eQTL discovery. Bioinformatics.

[49] Anders S, Huber W (2010). Differential expression analysis for sequence count data. Genome Biol..

[50] Anders S, Reyes A, Huber W (2012). Detecting differential usage of exons from RNA-seq data. Genome Res..

[51] Leroy PJRAM (2005). Robust Regression and Outlier Detection.

[52] Bennett DA (2018). Religious Orders Study and Rush Memory and Aging Project. J Alzheimers Dis.

[53] De Jager PL (2012). A genome-wide scan for common variants affecting the rate of age-related cognitive decline. Neurobiol. Aging.

[54] Boyle PA (2021). To what degree is late life cognitive decline driven by age-related neuropathologies?. Brain.

[55] Bennett DA, Schneider JA, Wilson RS, Bienias JL, Arnold SE (2004). Neurofibrillary tangles mediate the association of amyloid load with clinical Alzheimer disease and level of cognitive function. Arch. Neurol..

[56] Bennett DA (2003). Apolipoprotein E epsilon4 allele, AD pathology, and the clinical expression of Alzheimer's disease. Neurology.

