# Supplementary material for: Differential gene expression analysis based on linear mixed model corrects false positive inflation for studying quantitative traits
Source: Sci Rep. 2023 Oct 3;13:16570. doi: 10.1038/s41598-023-43686-7 (PMC10547771; doi:10.1038/s41598-023-43686-7)
Supplement: Supplementary file 1 — Supplementary Information. [file 41598_2023_43686_MOESM1_ESM.pdf]

Supplemental Information

Supplemental Text

RNA-Seq data of dorsolateral prefrontal cortex (DLPFC)

Samples were extracted using Qiagen's miRNeasy mini kit (cat. no. 217004) and the RNase free DNase Set (cat. no. 79254), and quantified by Nanodrop and quality was evaluated by Agilent Bioanalyzer. The Broad Institute's Genomics Platform performed RNA-Seq library preparation using the strand specific dUTP method (Levin et al., 2010) with poly-A selection (Adiconis et al., 2013). This method begins with poly-A selection followed by first strand specific cDNA synthesis, and then uses dUTP for second strand specific cDNA synthesis followed by fragmentation and Illumina adapter ligation for library construction. All analyzed samples met quality (Bioanalyzer RNA integrity (RIN) score >5) and quantity thresholds (5ug). Sequencing was performed on the Illumina HiSeq with 101bp paired-end reads. RNA-Seq data were processed by the ROS/MAP parallelized and automatic pipeline. The pipeline includes trimming the beginning and ending bases from each read, identifying and trimming adapter sequences from reads, detecting and removing rRNA reads, aligning reads to reference genome. Non-gapped aligner Bowtie was used to align reads to transcriptome reference and then RSEM was applied to estimate expression levels for all transcripts.

RNA-Seq data of three motor function related tissues

Tissue homogenization

For muscle tissue embedded in OCT, each piece was thawed in 3 ml ice-cold DPBS, then the thawed OCT was removed with a disposable plastic spatula. Frozen brain (supplementary motor

area), muscle, or spinal cord tissue was combined with DNA/RNA shield buffer (Zymo, R1100; 700 ul for brain/muscle, 300 ul for spinal cord) in 1.5-ml tubes containing 3-mm zirconium beads (Benchmark Scientific, D1032-30). Samples were then homogenized using a BeadBlaster at speed 6.5 for two 30-sec pulses separated by a 30-sec pause and stored at 4C overnight. Samples were then spun at 15,000 x g for 2 min to pellet insoluble material, and the supernatant (homogenate) was transferred to 1-ml Matrix tubes and stored at 4C (short-term) or -80C (long-term).

RNA extraction

RNA was extracted from 300 ul Shield homogenate using Chemagic RNA tissue kit (Perkin Elmer, CMG-1212) on a Chemagic 360 instrument. RNA-depleted homogenates were removed from the robot 30 min into the run and used for protein precipitation (see below). RNA was further purified and concentrated (Zymo, R1080) and RQN values calculated with a Fragment Analyzer total RNA assay (Agilent, DNF-471). RNA concentration was determined using Qubit broad range RNA assay (Invitrogen, Q10211) according to the manufacturer's instructions. RNA was normalized to 30 ng/ul for sequencing library prep.

Sequencing library preparation

300 ng total RNA was used as input for sequencing library generation and rRNA was depleted with RiboGold (Illumina, 20020599). A Zephyr G3 NGS workstation (Perkin Elmer) was utilized to generate TruSeq stranded sequencing libraries (Illumina, 20020599) with custom unique dual indexes (IDT) according to the manufacturer's instructions with the following modifications. RNA was fragmented for 4 minutes at 85°C. First strand synthesis was extended to 50 minutes. Size selection after adapter ligation was performed using a 0.8X ratio of AmpureXP beads.

Library size and concentrations were determined using an NGS fragment assay (Agilent, DNF-473) and Qubit ds DNA assay (Invitrogen, Q10211) respectively, according to the manufacturer's instructions. The modified protocol yielded libraries with an average insert size of around 330-370bp. Libraries were sequenced on a NovaSeq 6000 (Illumina) at 40-50M reads, 2x150bp paired-end.

RNA-Seq data processing

RNA-Seq data processing was implemented using two parallel pipelines, an RNA-seq QC pipeline, a gene/transcripts quantification pipeline. In the QC pipeline, the paired-end RNA-Seq data were first aligned by STAR v2.623 to a human reference (GRch38) with transcriptome annotations (gencode v27). CollectMultipleMetrics, CollectRnaSeqMetrics and MarkDuplicates in Picard toolkits were applied to the aligned bam files to assess the quality of RNA-Seq data. In the quantification pipeline, transcript raw counts and TPM were calculated by Kallisto (v0.46). Transcript sequencing files (fasta) were downloaded from Gencode (v27).

Supplemental References

Adiconis, X., Borges-Rivera, D., Satija, R., DeLuca, D. S., Busby, M. A., Berlin, A. M., . . . Levin, J. Z. (2013). Comparative analysis of RNA sequencing methods for degraded or low-input samples. *Nature Methods, 10*(7), 623-629. doi:10.1038/nmeth.2483

Levin, J. Z., Yassour, M., Adiconis, X., Nusbaum, C., Thompson, D. A., Friedman, N., . . . Regev, A. (2010). Comprehensive comparative analysis of strand-specific RNA sequencing methods. *Nature Methods, 7*(9), 709-715. doi:10.1038/nmeth.1491

Supplemental Tables

	Tissue	Sample Size	NCI	MCI	Alzheimer's Dementia	Other Dementia
Discovery Data	DLPFC	632	200	168	252	12
Validation Data	DLPFC	588	200	120	260	7
	SMA	234	87	66	79	2
	Spinal cord	232	86	63	81	2
	Muscle	268	102	78	86	2

Supplemental Table 1. Sample sizes and cognition diagnostic features of samples in the discovery and validation datasets. Participants are not overlapped between discovery and validation data sets, but largely overlapped for SMA, spinal cord, and muscle tissues in the validation data. DLPFC: dorsolateral prefrontal cortex. SMA: supplemental motor area. NCI: no cognitive impairment. MCI: mild cognitive impairment.

	Variable (range)	Mean (SD) or N (%)
**Demographics**	Age at death (years) (65.91-104.20)	90.4 (6.48)
	Male	162 (27.6%)
	Postmortem Interval (PMI, hours) (0.87-115.75)	9.67 (8.13)
	MAP participants	382 (65.0%)
**Cognitive AD Trait**	Rate of cognitive decline (-0.50, 0.24)	-0.01 (0.1)
**AD Neuropathologic Change (AD-NC) Traits**	β-Amyloid (0.00, 22.94)	4.36 (4.06)
	Tangle density (0.00, 61.01)	8.01 (9.63)
	Global AD pathology burden (0.00, 2.81)	0. 77 (0.64)

Supplemental Table 2. Characteristics of the validation RNA-Seq data (n=588) of DLPFC samples.

Quantitative Traits	Discovery Data	Validation Data			
	DLPFC	DLPFC	SMA	Spinal cord	Muscle
Cognitive decline	565	541	200	197	234
β-Amyloid	587	574	104	100	115
Tangle density	587	580	108	104	121
Global AD pathology burden	592	588	119	115	134

Supplemental Table 3. Sample sizes for DGE analyses of all quantitative AD traits in both discovery and validation datasets.

	Variable<br>(range)	Mean (SD) or N (%)
**Demographics**	Age at death (years)<br>(71.7-108.3)	91.5 (5.89)
	Male	163 (27.6%)
	Postmortem Interval (PMI, hours)<br>(3.8-43.9)	8.6 (5.12)
**Cognitive AD Trait**	Rate of cognitive decline<br>(-0.32, 0.24)	0.01 (0.09)
**AD Neuropathologic Change (AD-NC) Traits**	β-Amyloid<br>(0.00, 14.32)	3.06 (3.67)
	Tangle density<br>(0.00, 42.31)	5.39 (8.16)
	Global AD pathology burden<br>(0.00, 2.39)	0.57 (0.59)

Supplemental Table 4. Characteristics of the validation RNA-Seq datasets of three motor function related tissue types — SMA, spinal cord, and muscle.

Supplemental Figures

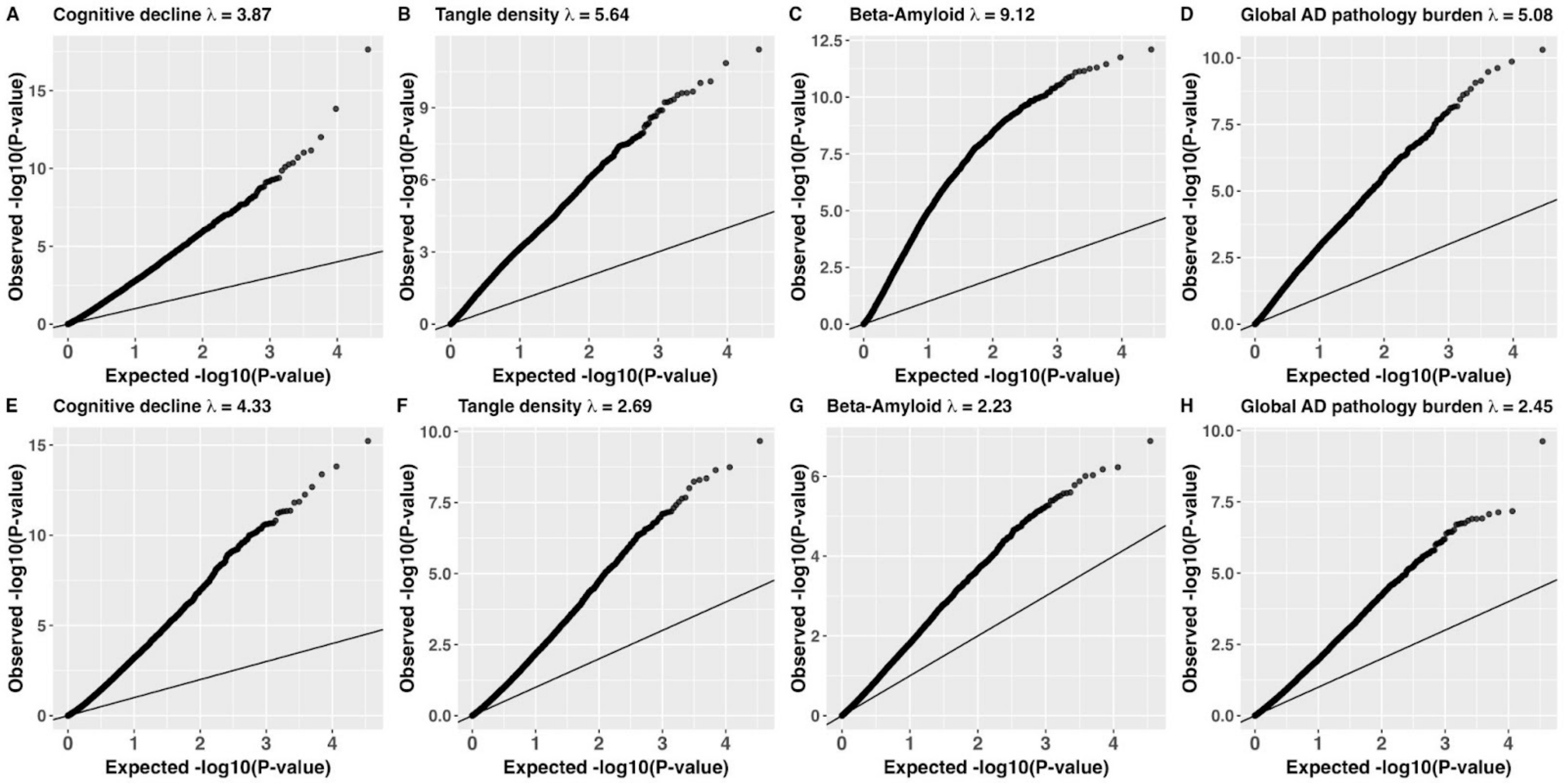

Supplemental Fig 1. QQ-plots and genomic control factors (λ) of DGE results of cognitive decline and three AD pathologies by robust regression with the discovery RNA-Seq data of DLPFC tissue (A-D) and the validation RNA-Seq data of DLPFC tissue (E-H).

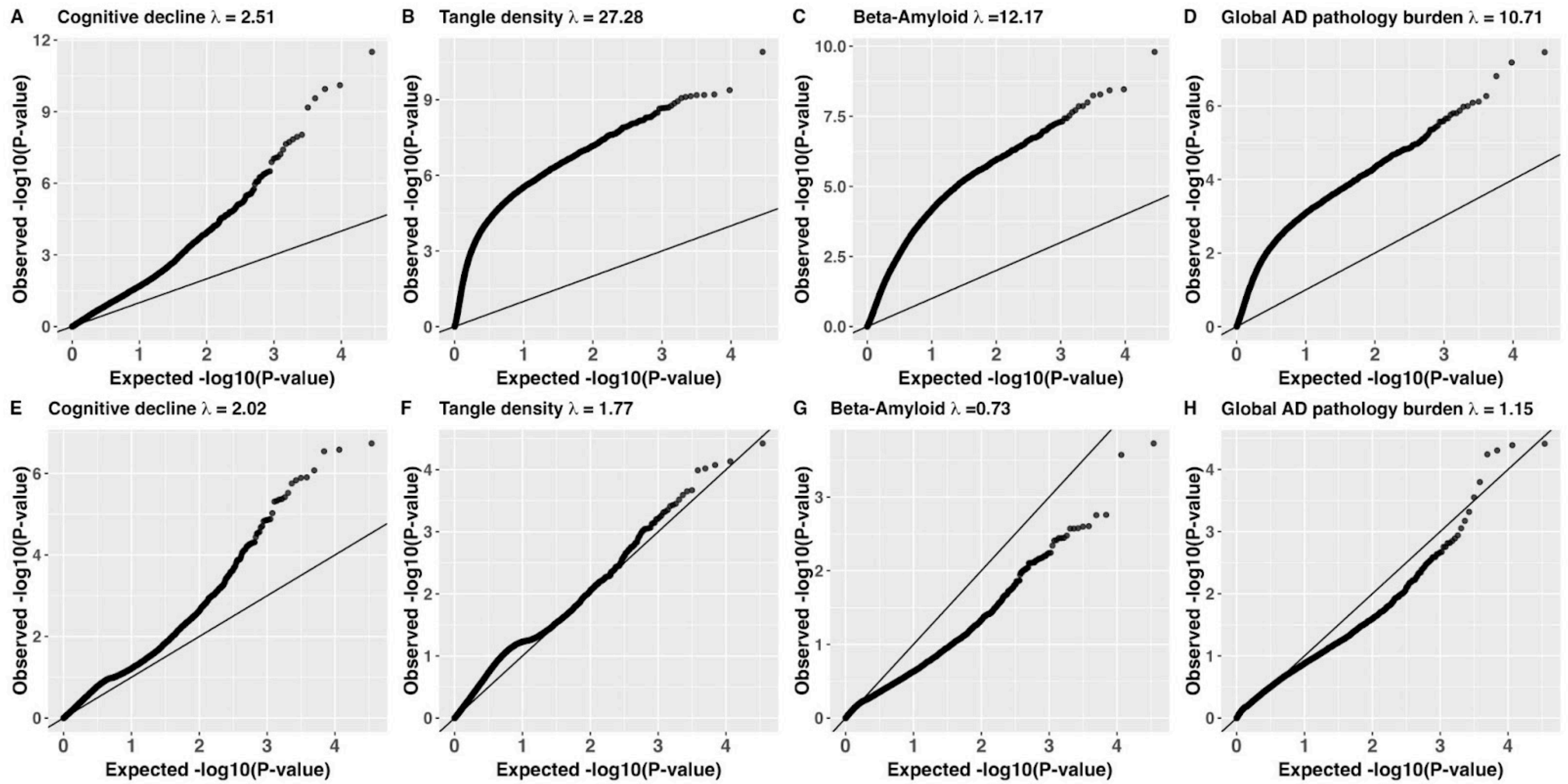

Supplemental Fig 2. QQ-plots and genomic control factors (λ) of DGE results by Voom with the discovery RNA-Seq data of DLPFC tissue (A-D) and the validation RNA-Seq data of DLPFC tissue of cognitive decline and three AD pathologies (E-H).

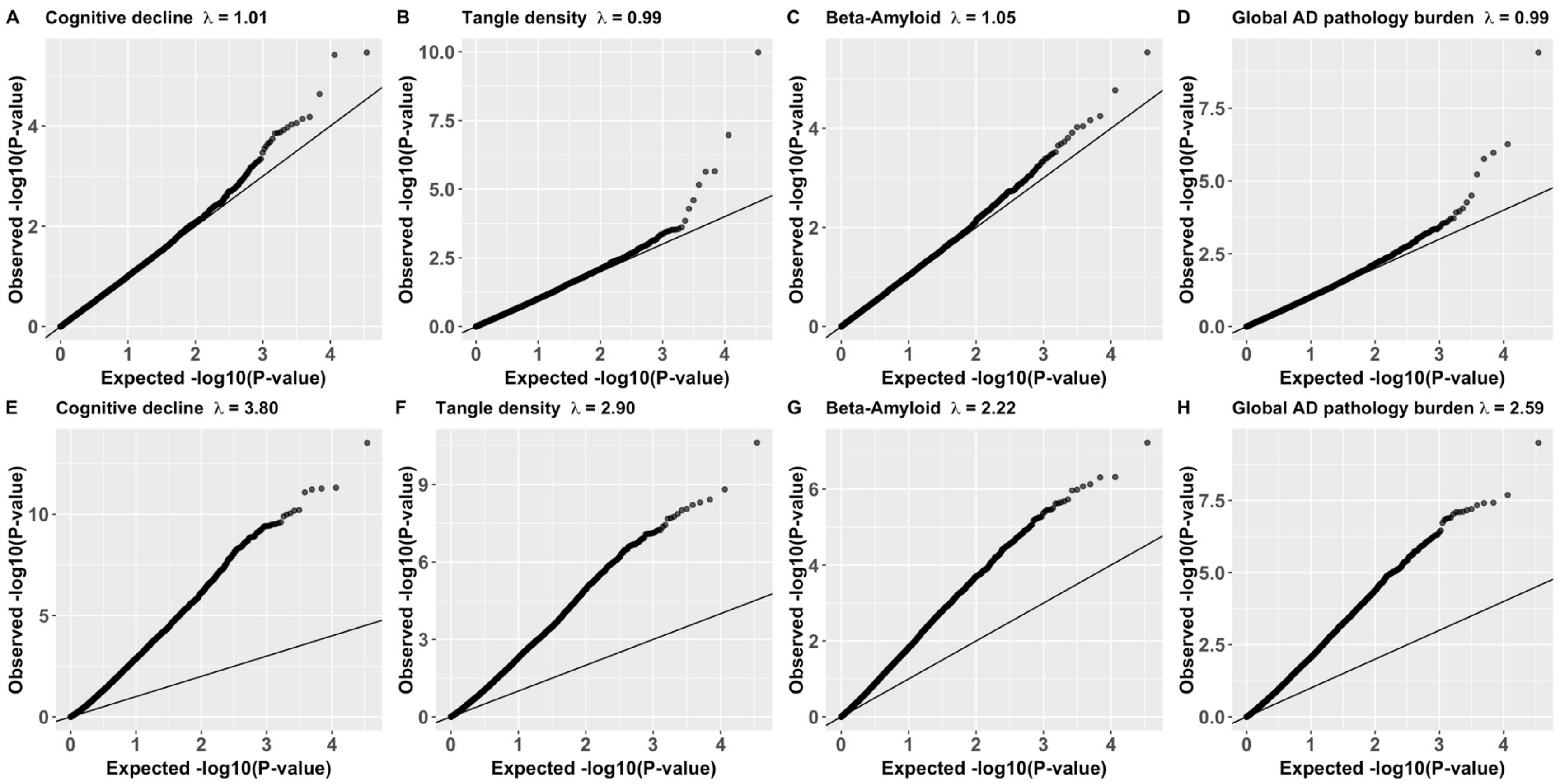

Supplemental Fig 3. QQ-plots and genomic control factors (λ) of DGE results of cognitive decline and three AD-NC traits by LMM (A-D; first row) and standard linear regression model based methods (E-H; second row), with the validation RNA-Seq data of DL-PFC.

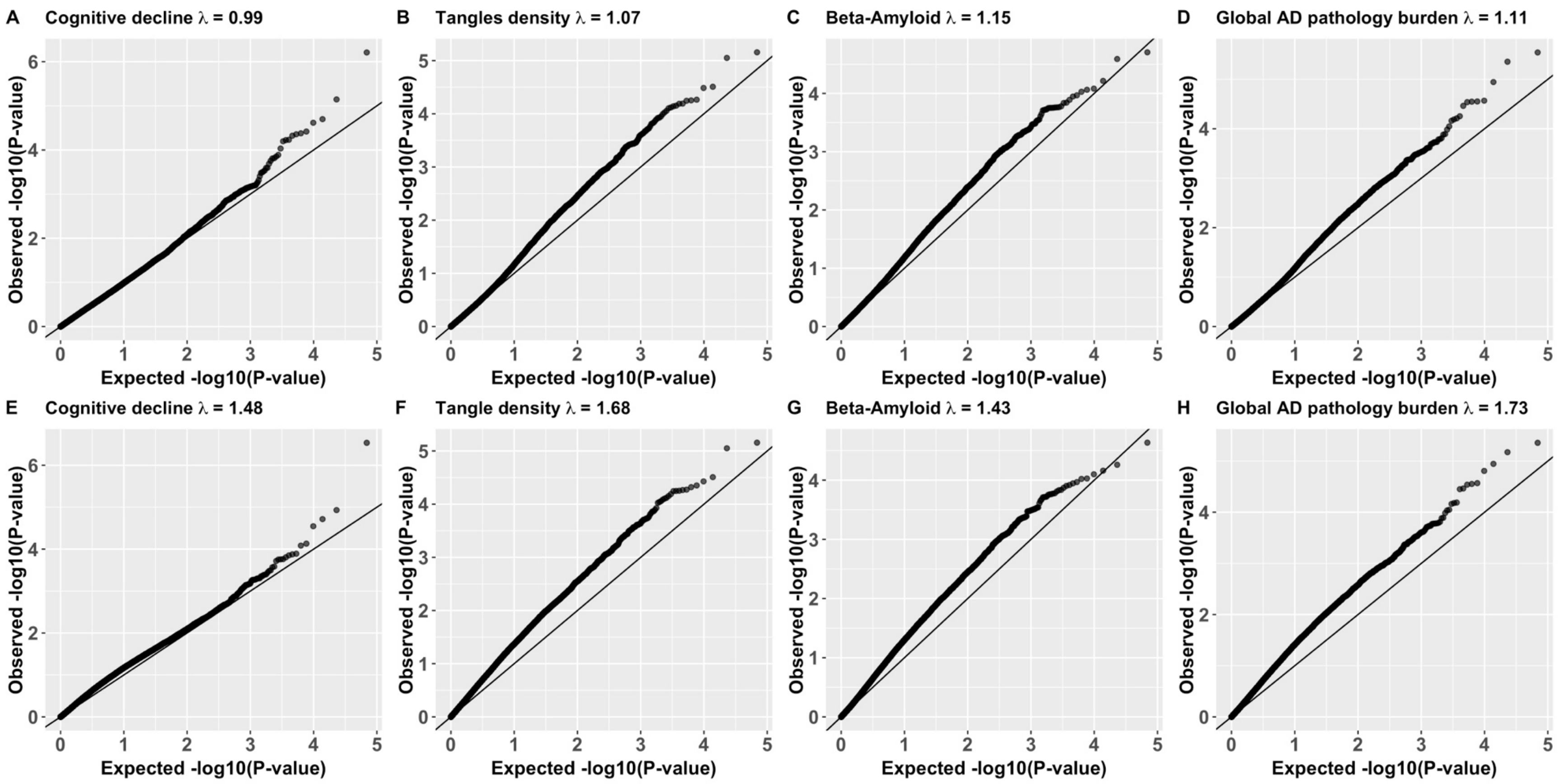

Supplemental Fig 4. QQ-plots and genomic control factors (λ) of DGE results of cognitive decline and three AD-NC traits by LMM (A-D; first row) and standard linear regression model based methods (E-H; second row), with the validation RNA-Seq data of SMA.

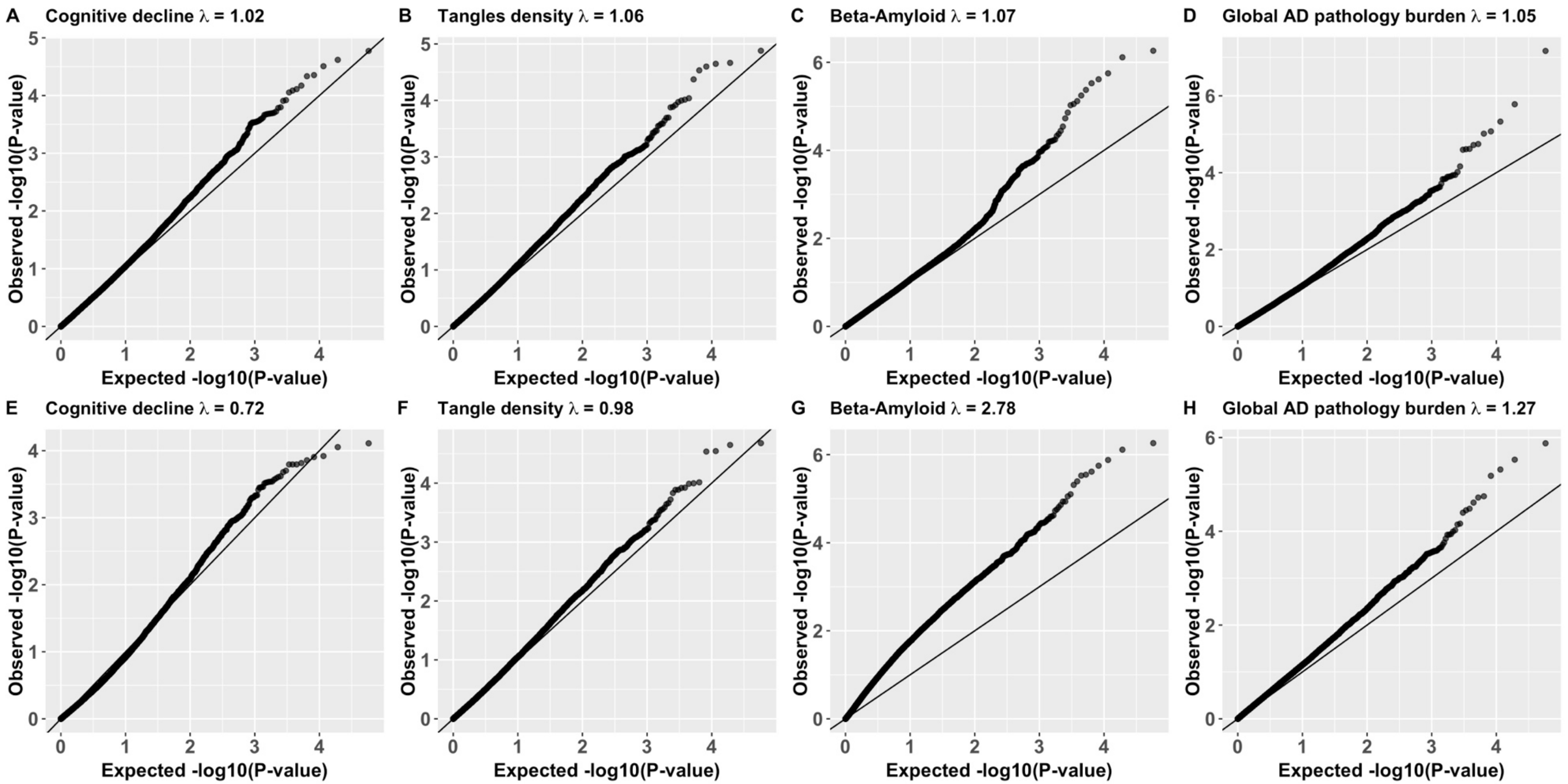

Supplemental Fig 5. QQ-plots and genomic control factors (λ) of DGE results of cognitive decline and three AD-NC traits by LMM (A-D; first row) and standard linear regression model based methods (E-H; second row), with the validation RNA-Seq data of muscle.

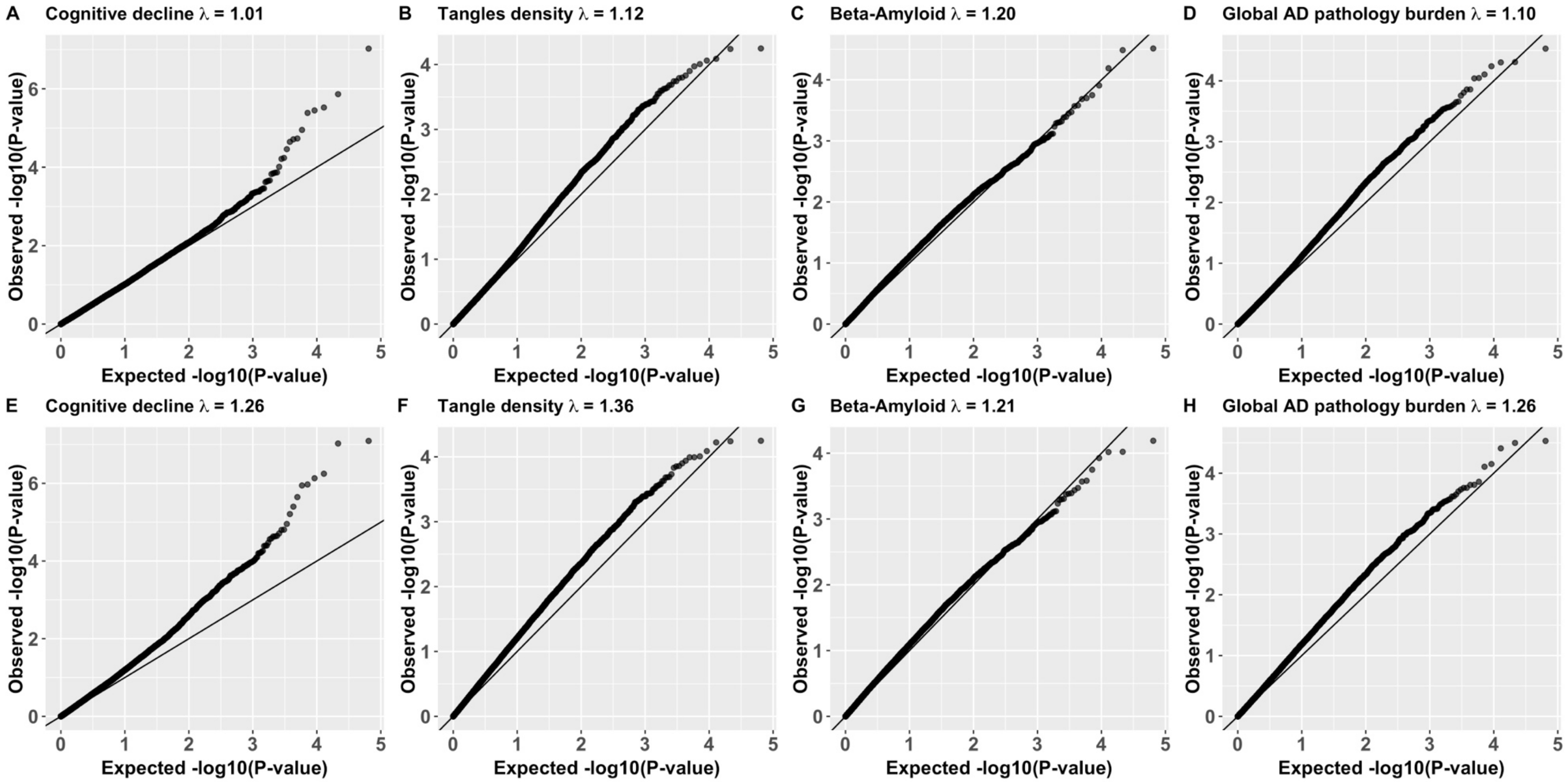

Supplemental Fig 6. QQ-plots and genomic control factors (λ) of DGE results of cognitive decline and three AD-NC traits by LMM (A-D; first row) and standard linear regression model based methods (E-H; second row), with the validation RNA-Seq data of spinal cord.

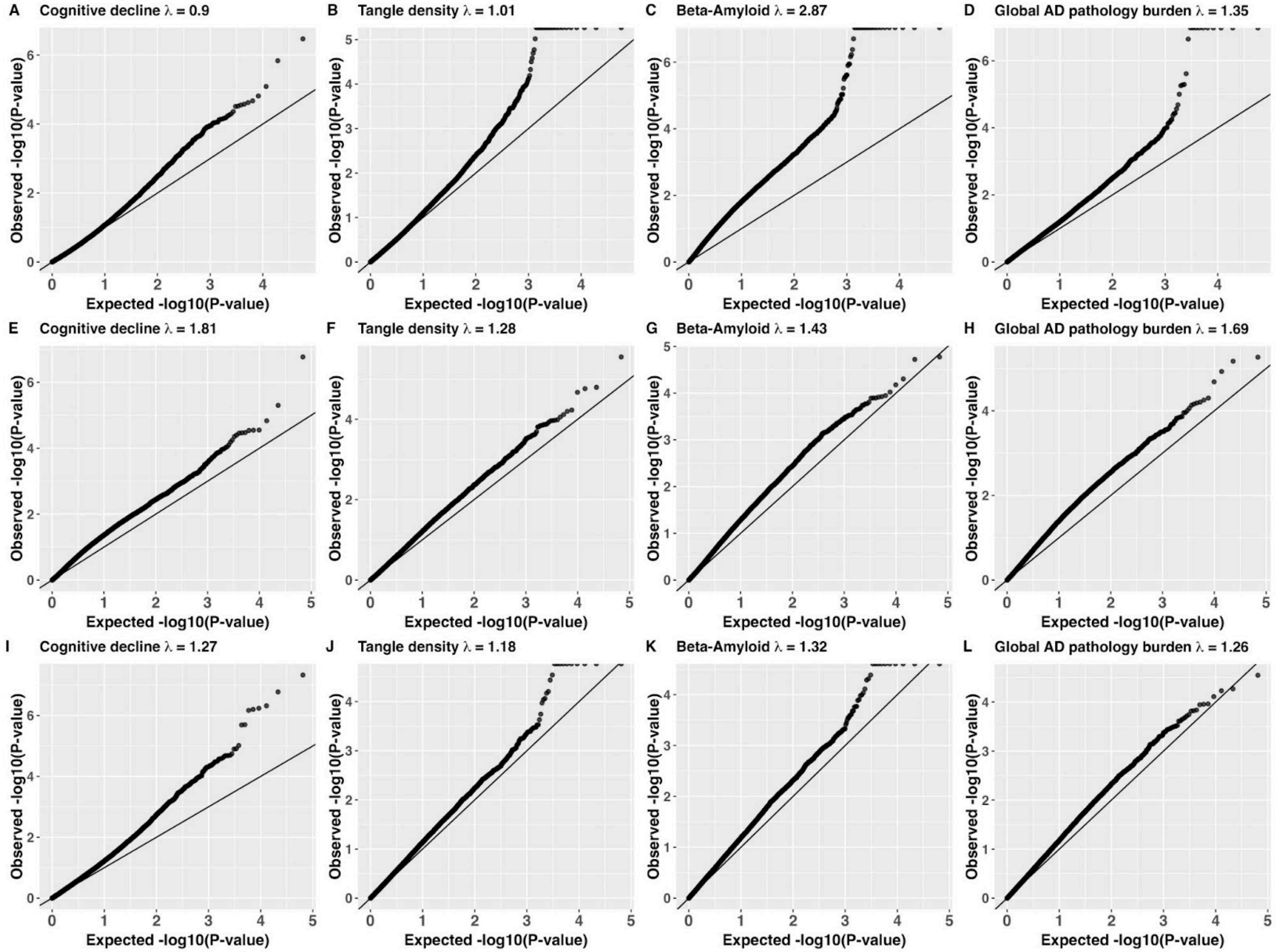

Supplemental Fig 7. QQ-plots and genomic control factors (λ) of DGE results by robust regression with the validation RNA-Seq data of muscle (A-D), validation RNA-Seq data of SMA (E-H) and validation RNA-Seq data of spinal cord (I-L).

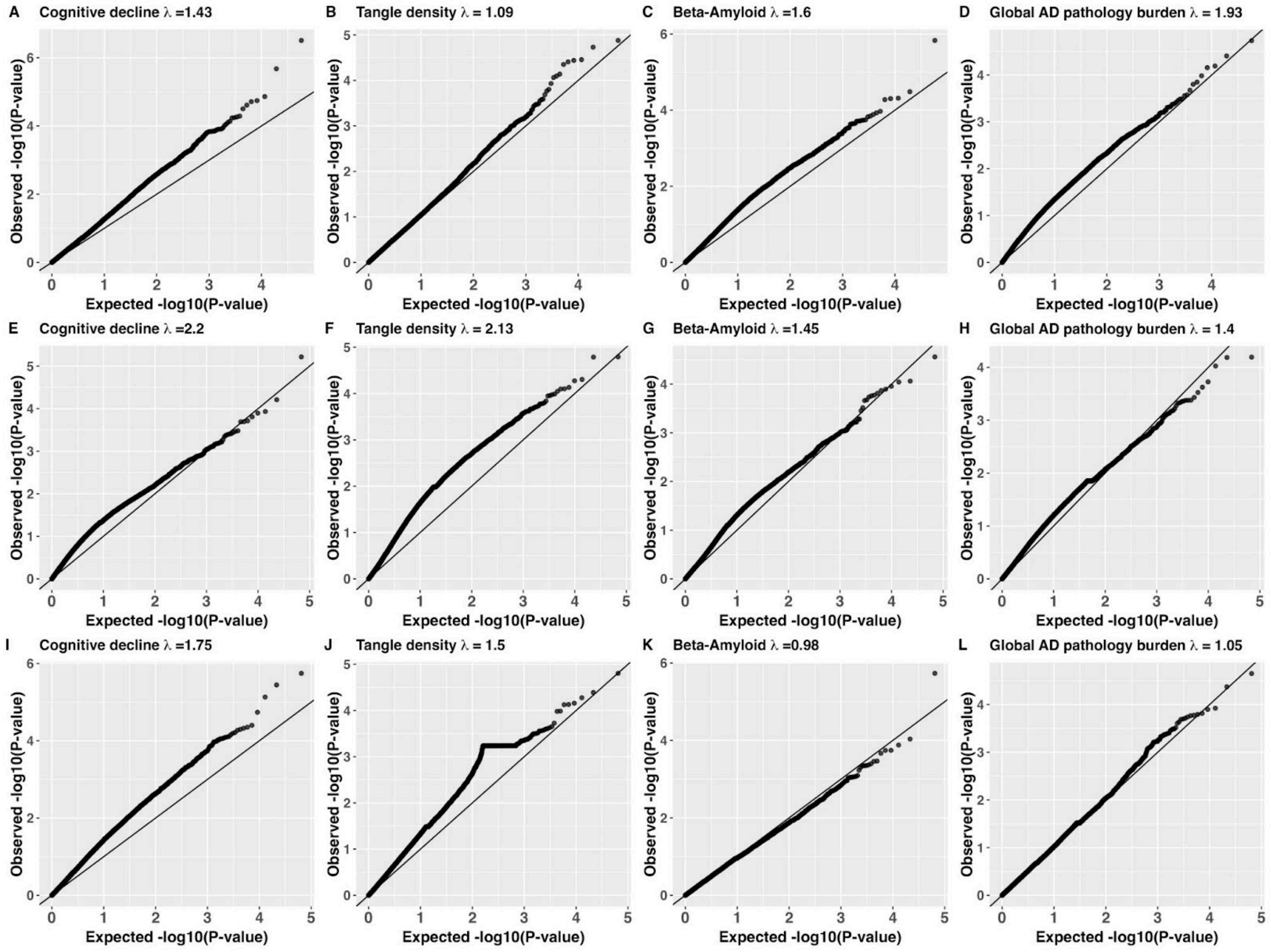

Supplemental Fig 8. QQ-plots and genomic control factors (λ) of DGE results by Voom with the validation RNA-Seq data of muscle (A-D), validation RNA-Seq data of SMA (E-H) and validation RNA-Seq data of spinal cord (I-L).

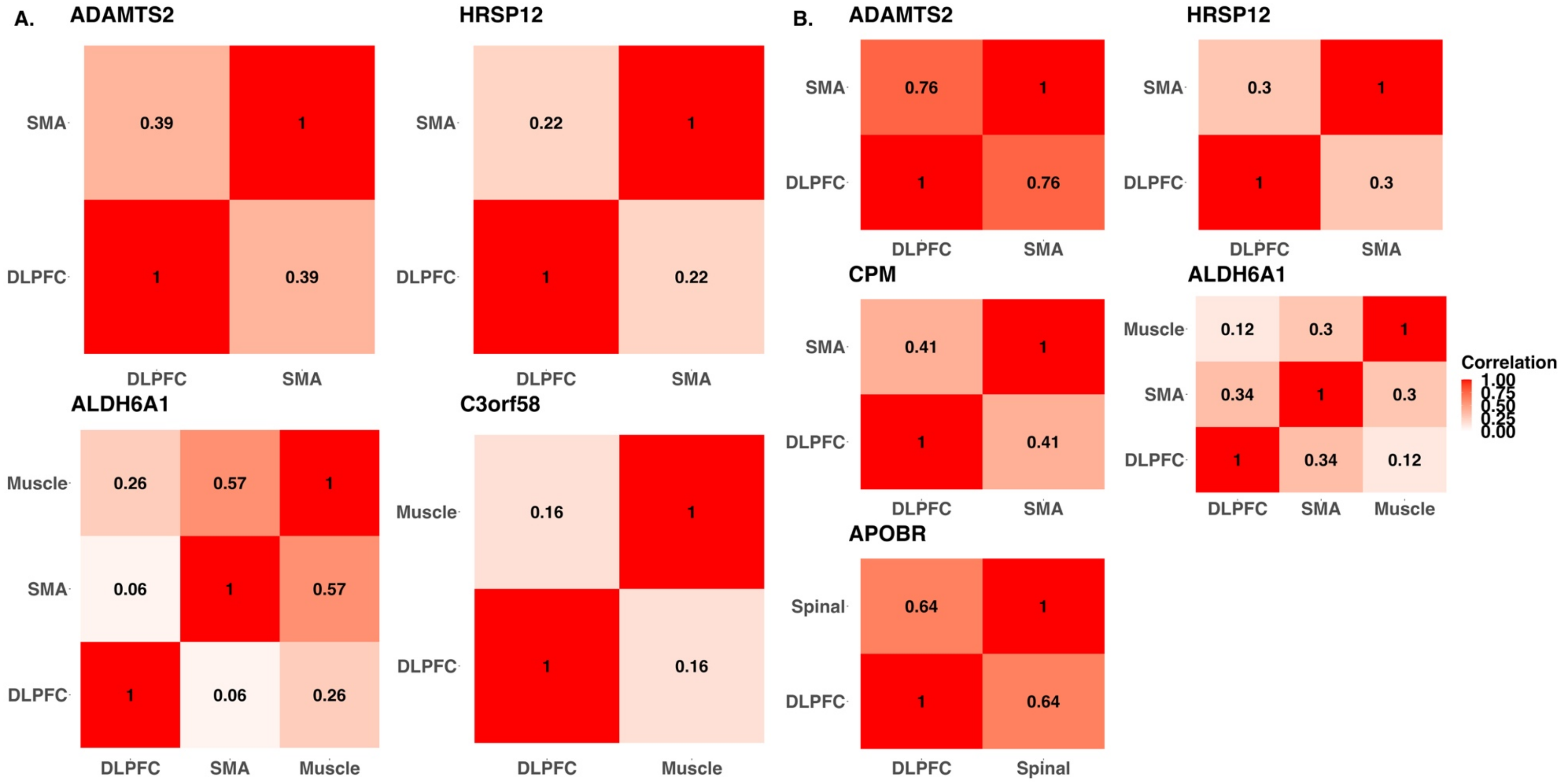

Supplemental Fig 9. The correlation heatmaps of the differentially expressed genes identified in the discovery RNA-Seq data of DLPFC that were replicated with cognitive decline (A) and global AD pathology (B) traits in SMA, spinal cord, and muscle tissues.

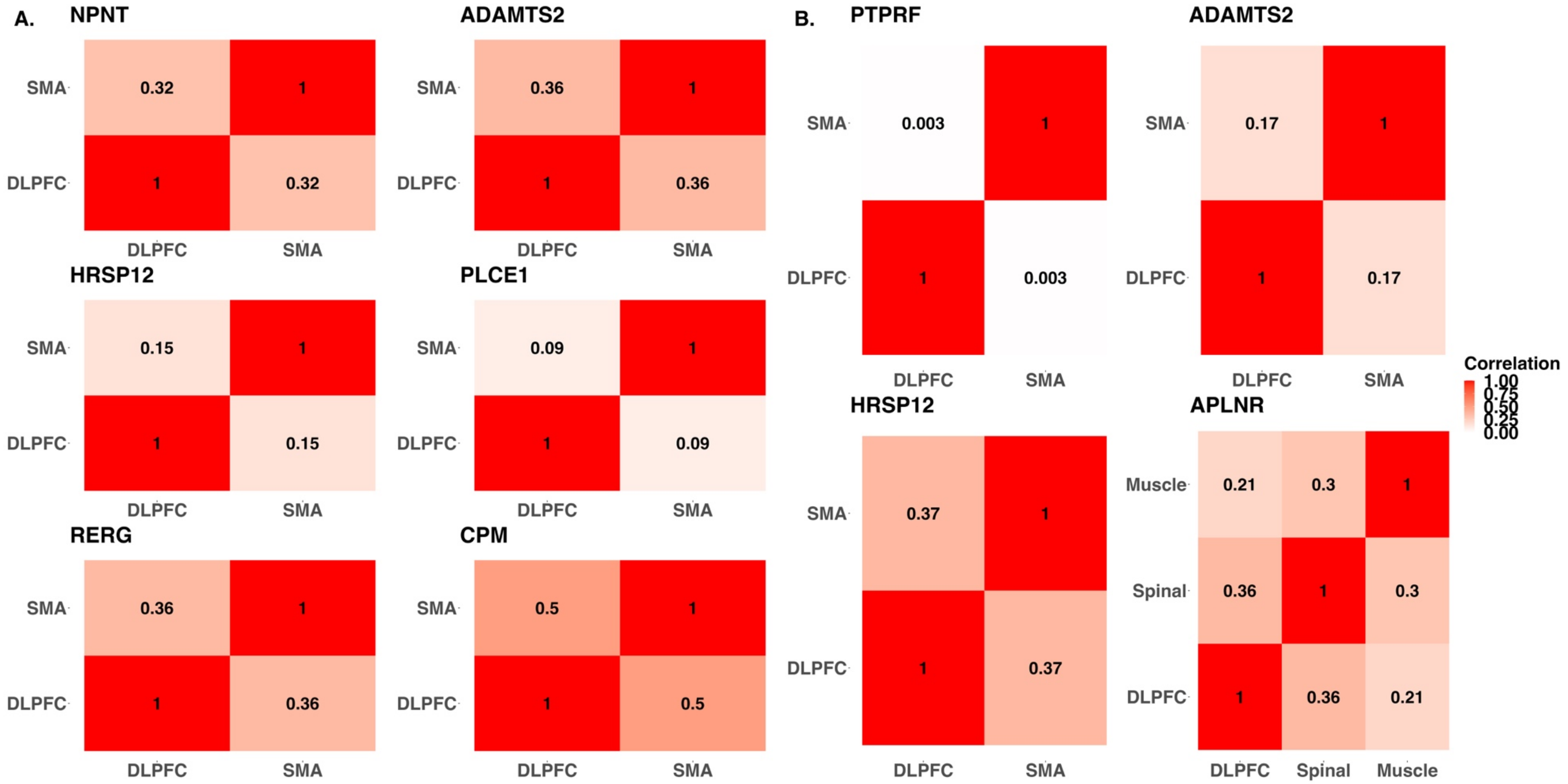

Supplemental Fig 10. The correlation heatmaps of the differentially expressed genes identified in the discovery RNA-Seq data of DLPFC that were replicated with tangle density (A) and β-Amyloid (B) traits in SMA, spinal cord, and muscle tissues.
